# Eliciting antitumor immunity via therapeutic cancer vaccines

**DOI:** 10.1038/s41423-025-01316-4

**Published:** 2025-07-09

**Authors:** Kun Peng, Xiaoxue Zhao, Yang-Xin Fu, Yong Liang

**Affiliations:** 1 Center for Cancer Biology, School of Basic Medical Sciences, Tsinghua University, Beijing, China; 2 State Key Laboratory of Molecular Oncology, Tsinghua University, Beijing, China; 3 Changping Laboratory, Beijing, China

**Keywords:** Tumor-associated antigen, Neoantigen, Vaccine, Cancer immunotherapy, Tumour vaccines, Tumour immunology

## Abstract

Therapeutic cancer vaccines aim to expand and activate antigen-specific T cells for the targeted elimination of cancer cells. While early clinical trials faced challenges due to suboptimal antigen-specific T-cell activation, recent advancements in antigen discovery and vaccine platform engineering have revitalized the field. This review provides a comprehensive overview of key tumor antigens, including tumor-associated antigens, viral oncoprotein antigens, neoantigens, and cryptic antigens, with a focus on their immunogenicity and therapeutic potential. Advances in our understanding of traditional cancer vaccination targets, in conjunction with the timely identification of novel antigen epitopes, have facilitated the strategic selection of vaccination targets. We also discuss the evolution of cancer vaccine platforms—spanning peptide-based formulations to advanced mRNA vectors—emphasizing innovative strategies to optimize antigen delivery efficiency and adjuvant effects. Efficient antigen delivery and adjuvant selection overcome immune tolerance and tumor-induced immunosuppression. Furthermore, we examine recent clinical trial data and emerging combination approaches that integrate cancer vaccines with other immunotherapies to increase efficacy. While significant progress has been made, challenges remain in improving vaccine-induced T-cell responses, overcoming immune suppression, and translating these advances into effective clinical interventions. Addressing these hurdles will be critical for realizing the full potential of cancer vaccines in immunotherapy.

## Introduction

Immunotherapy has emerged as a strategy to reprogram the immune system to control tumors, fundamentally changing cancer treatment paradigms. Immune checkpoint inhibitors (ICIs), which block cancer-derived immune-suppressive signals, represent the most successful immunotherapy approach to date [1]. Long-term complete response has been achieved in specific subsets of patients with certain cancers [2]. However, most cancer patients either fail to respond or experience acquired resistance, underscoring the urgent need for new cancer immunotherapy strategies [3].

The tumor microenvironment (TME) involves multiple immune-suppressive mechanisms that inhibit antitumor immunity, allowing cancer cells to evade immune detection [4]. The density of tumor-infiltrating T cells is correlated with patient prognosis. Primary and acquired resistance to ICIs often results from insufficient antitumor T cells [3]. To address this problem, numerous immunotherapy strategies have been developed to expand preexisting and prime de novo tumor-specific T cells to reactivate antitumor immune responses. A promising approach for providing more de novo antitumor T cells is therapeutic cancer vaccination [5]. This strategy focuses on identifying tumor antigens and designing potent vaccine formulations capable of delivering both antigens and adjuvant signals to activate antigen-presenting cells (APCs) for efficient T-cell priming and reactivation inside lymphoid tissues [6]. Earlier clinical trials of cancer vaccines relied on tumor-associated antigens (TAAs) that were overexpressed in cancer cells and utilized traditional vaccine platforms, including proteins, DNA, viral vectors, and whole cancer cells [7]. However, these trials largely failed because of self-tolerance to TAAs, poor adjuvants, toxicity, and the suppressive nature of the tumor microenvironment. Recent technological advancements have revolutionized cancer vaccination by targeting tumor-specific antigens (TSAs).

Cancer is characterized by genomic instability, with numerous point mutations and chromosomal alterations accumulating during tumor progression [8]. These genetic mutations can give rise to altered proteins, which are recognized by the immune system as “nonself” antigens or neoantigens, potentially triggering cellular immune responses that counteract tumor growth [9]. Tumor mutation burdens (TMBs) are generally correlated with the ICI response rate. With rapid advances in genomic sequencing and antigen prediction across diverse HLA complexes, potential immunogenic neoantigens can now be identified with certain precision [10]. Artificial intelligence (AI) may further accelerate the identification of genuine immunogenic neoantigens. These epitopes are tumor-specific and nontolerant to the host immune system. Furthermore, novel vaccine platforms have been developed to enhance antitumor T-cell responses. This review explores tumor antigens and adjuvants, detailing the development of diverse vaccination platforms, including peptide and mRNA vaccines. Optimizing antigen selection and vaccine formulation holds the potential to revitalize cancer vaccination as a treatment strategy capable of stimulating immune responses in ICI-resistant patients.

## Antigen selection for the design of cancer vaccines

**Fig. 1:**
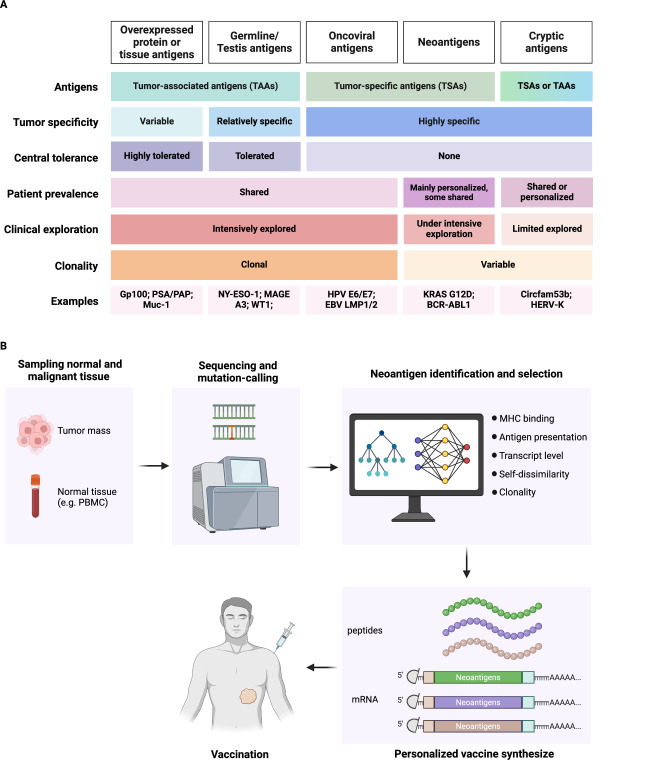
Cancer Antigen Categories and Personalized Neoantigen Vaccine Design. **A** Tumor antigens can be roughly classified into five categories: tumor-associated antigens (TAAs), cancer/testis antigens, oncoviral antigens, neoantigens, and cryptic antigens. The table summarizes the key properties of each category, including tumor specificity, central tolerance, patient prevalence, clonality among cancer cells, and clinical exploration. Notable examples are also provided. **B** Clinical procedure for designing personalized neoantigen vaccines. Tumor and normal tissue (e.g., PBMC) biopsies are obtained from patients and sent for next-generation sequencing (NGS) and mutation calling. The identified nonsynonymous mutations are evaluated for MHC binding, antigen presentation, transcript levels, self-dissimilarity, and clonality. Selected neoantigen epitopes are synthesized into peptide or mRNA vaccines for patient treatment

The development of an effective vaccine for tumor rejection requires the identification of reliable tumor antigens that are highly expressed in tumor tissues and capable of eliciting robust antigen-specific T-cell responses. On the basis of parental gene expression, tumor antigens are broadly categorized into tumor-associated antigens (TAAs) and tumor-specific antigens (TSAs) (Fig. 1A). TAAs are self-proteins that are overexpressed in cancer cells, including tissue-specific antigens and developmentally associated germline/cancer testis antigens. TSAs are antigens that are restricted to tumors and are rarely found in normal tissues. They can arise from genetic alterations (neoantigens), oncogenic viruses involved in oncogenic transformation (oncoviral antigens), expression of endogenous retroviral elements, or presumably noncoding sequences within tumors (cryptic antigens).

Some tumor antigens, referred to as shared or universal antigens, are present in multiple patients and serve as promising targets for universal vaccines. In contrast, personalized antigens are unique to specific cancer cells in individual patients and have garnered attention with advances in the rapid identification of neoantigens. In the following sections, we summarize the characteristics of different tumor antigen types, outline key experimental pipelines for formulating neoantigen vaccines in the clinic, and discuss critical considerations for identifying and selecting neoantigens for individualized therapeutic cancer vaccines.

### Tumor-associated antigens

Some tumor types highly express tissue-specific antigens, which have been proven to be immunogenic and tested in clinical trials [7, 11]. Human epidermal growth factor receptor 2 (HER2/Neu), a member of the EGFR kinase family, is overexpressed in approximately 20% of breast cancers and some gastrointestinal and ovarian tumors [12]. This antigen is commonly targeted with therapeutic anti-HER2 antibodies or antibody‒drug conjugates (ADCs) [13]. A peptide vaccine comprising both HLA-I- and HLA-II-binding HER2 peptides has induced durable CD8 ^+^ T-cell responses in patients, demonstrating the immunogenicity of this antigen [14]. Gp100, which is expressed in melanoma, has been extensively studied as a target for both vaccine and adoptive cell transfer (ACT) therapy [15, 16]. Phase III clinical trials have shown that gp100 peptide vaccination can improve the overall response rate (ORR) when it is combined with high-dose IL-2 but not with ipilimumab [15, 17]. However, gp100 vaccine monotherapy has limited therapeutic efficacy, possibly because of central tolerance mechanisms that eliminate high-affinity gp100-recognizing T cells. In contrast, ACT of engineered T cells with high affinity for gp100 has demonstrated superior antitumor immunity in melanoma models but is associated with autoimmune side effects [18]. Prostatic acid phosphatase (PAP) and prostate-specific antigen (PSA) are expressed on prostate epithelial cells [19]. A phase III trial of sipuleucel-T, an autologous GM-CSF-stimulated monocyte mixture pulsed with PAP, demonstrated a 4-month survival benefit over control vaccination, leading to FDA approval for prostate cancer [20]. However, peptide- or virus-based PAP and PSA vaccines failed to demonstrate a survival benefit in clinical trials, highlighting the need to improve vaccination platforms to increase T-cell activation [21]. Other antigens, such as p53, MUC-1, IDO1, survivin, and PD-L1, are overexpressed in various tumor types and have been evaluated in clinical trials for their ability to induce antigen-specific T-cell responses [22–26].

Germline/testis antigens, which are expressed primarily in fetal tissues and testes, can be re-expressed in malignant tissues, contributing to carcinogenesis and immune evasion. Wilms’ tumor protein (WT1), a developmental transcription factor involved in oncogenesis, is overexpressed in most acute myeloid leukemias (AMLs), breast cancers, and Wilms’ tumors [27]. The National Cancer Institute (NCI) has ranked WT1 as a top candidate for cancer vaccination [28]. Vaccination with a heteroclitic WT1 peptide (Galinpepimut-S) with enhanced HLA affinity induced T-cell responses in most AML patients in a phase II trial, supporting further exploration of this antigen [29]. New York-esophageal cancer 1 (NY-ESO-1), a cancer-testis antigen, is highly expressed in synovial sarcomas and variably expressed in melanoma, ovarian and esophageal cancers [30]. Spontaneous antigen-specific T-cell responses against NY-ESO-1 are frequently observed, indicating its immunogenicity [31, 32]. However, multiple clinical trials have failed, likely because of suboptimal vaccination designs and heterogeneous antigen expression in cancers [33–35]. In synovial sarcoma, where NY-ESO-1 expression is homogeneous, adoptive transfer of NY-ESO-1-specific T cells has yielded impressive clinical responses [36]. A state-of-the-art RNA vaccine encoding NY-ESO-1 and three other TAAs has demonstrated promising clinical outcomes, suggesting that potent vaccination platforms targeting multiple antigens can improve efficacy and prevent antigen escape [37]. Melanoma-associated antigen 3 (MAGE-A3), a cancer-testis antigen with antiapoptotic functions, is preferentially expressed in melanoma, non-small cell lung cancer (NSCLC), and breast cancer [38]. A TLR9 agonist-adjuvanted MAGE-A3 vaccine induced CD4^+^ T-cell and antibody responses in a phase III trial but failed to improve disease-free survival [39, 40]. In a large randomized trial, a multivalent melanoma vaccine containing MAGE-A3, Melan-A, gp100, and tyrosinase (seviprotimut-L) improved outcomes in subsets of younger patients and those with ulcerated primary melanomas, emphasizing the importance of patient selection for vaccination [41].

Central tolerance mechanisms may delete high-affinity TCR clones against TAAs or induce regulatory T cells toward tissue-specific antigens. Thus, while TAAs are potential candidates for patients with a low tumor mutational burden (TMB), their efficacy is limited by immune tolerance, poor immunogenicity, and tumor heterogeneity. Potent adjuvants may overcome tolerance but can cause detrimental autoimmunity due to the expression of the antigen in normal tissues. Therefore, the development of TAA-based vaccines must balance tumor-specific T-cell responses with autoimmune toxicity.

### Tumor-specific antigens

Currently, more efforts are focused on developing cancer vaccines targeting shared TSAs or personalized neoantigens, which could circumvent immune tolerance and elicit potent tumor-specific T-cell responses with improved safety.

#### Oncoviral antigens

Some oncogenic viruses, such as Epstein–Barr virus (EBV) and human papillomavirus (HPV), can drive oncogenic transformation [42]. These viral antigens are not subject to central tolerance, making them ideal targets for vaccination. EBV-encoded latent membrane proteins (LMP1 and LMP2) are expressed in natural killer (NK)–T-cell lymphoma, nasopharyngeal carcinoma, and other malignancies [43]. Adoptive transfer of autologous cytotoxic T cells targeting LMPs has resulted in durable clinical responses in lymphoma patients, spurring interest in clinical trials for EBV vaccines [44]. An mRNA vaccine encoding truncated domains with multiple T-cell epitopes from EBV proteins has demonstrated cellular immunity and prolonged survival in preclinical models [45]. Additionally, a modified vaccinia Ankara (MVA) virus expressing an Epstein–Barr nuclear antigen (EBNA)–LMP2 fusion protein increased CD4^+^ and CD8^+^ T-cell responses in EBV-positive patients, encouraging further large-scale clinical studies [46].

HPV E6 and E7 are viral TSAs that inhibit the tumor suppressors p53 and retinoblastoma protein (pRb), thereby promoting proliferation and tumorigenesis in squamous epithelial cells [47]. Nearly 60% of oropharyngeal cancers and almost all cervical cancers are positive for E6 and E7 antigens, making them highly attractive targets for vaccination [48]. Both mRNA-based and peptide-based vaccines have elicited robust antigen-specific T-cell responses in preclinical HPV mouse tumor models [49, 50]. In clinical studies, synthetic long peptides (SLPs), DNA, and viral-vector vaccines have demonstrated promising efficacy in treating cervical intraepithelial neoplasia, an early stage of carcinogenesis [51–53]. However, further research is needed to evaluate the effectiveness of E6/E7-based vaccines in more advanced-stage tumors, potentially in combination with other therapies to achieve clinical benefit.

### Neoantigens

Neoantigens are a subset of TSAs arising from tumor-specific genetic mutations. Neoantigen epitopes are absent in normal tissues, making them exempt from immune tolerance and more recognizable by T cells [6, 10, 54–57]. Single-cell RNA sequencing (scRNA-seq) across various cancers has suggested that most intratumoral T cells recognize neoantigen epitopes rather than TAAs [58–62], underscoring their superior immunogenicity and potential for therapeutic vaccination.

#### Molecular characterization of neoantigens

Genomic mutations, such as single-nucleotide variants (SNVs), insertions and deletions (indels), and gene fusions, can generate immunogenic neoantigens [10]. SNVs involve the substitution of single nucleotides within the genome and are the most abundant mutation type in cancers [63]. The SNV burden is correlated with the clinical efficacy of ICI therapies [64, 65]. Early studies focused on SNV-derived neoantigens, demonstrating primary clinical efficacy in various tumor models. Some common SNVs, such as the KRAS G12 and the IDH1 R132H mutations, are immunogenic and are shared across cancer patients [66, 67]. However, most SNVs are patient-specific and limited by tumor heterogeneity [68]. Indels can introduce frameshift sequences unrelated to the original protein with potential immunogenicity [69]. Clinical studies have shown that indel-derived neoantigens exhibit higher HLA-binding affinity than SNV-derived epitopes do. Moreover, shared immunogenic polyepitopes from indels have been observed in microsatellite-unstable tumors [70]. Gene fusions occur through chromosomal rearrangements, creating new sequences. Although gene fusion-derived antigens are rare compared with SNVs and indels [71], a well-known example is the BCR–ABL1 fusion gene, which is found in ~90% of patients with chronic myelogenous leukemia (CML) [72]. In addition to genomic mutations, neoantigens can also arise from transcriptional and posttranscriptional alterations, such as alternative splicing and A-to-I RNA editing [73–80]. However, these neoantigens require further validation in clinical trials.

Although some neoantigens are shared among a small subset of patients, most neoantigens are highly individualized [68, 69, 81]. The development of personalized neoantigen vaccines is therefore necessary. However, this approach faces significant challenges, as the immunogenicity of neoepitopes varies considerably, with only 1–2% of identified nonsynonymous mutations in tumors recognized by T cells [82]. Advancing neoantigen vaccines requires sophisticated experimental methods, AI-driven analysis, and computational pipelines to identify tumor-expressed mutated peptides and assess their vaccine potential, offering a promising yet complex and resource-intensive approach for cancer immunotherapy.

#### Identification of neoantigen epitopes

Identifying mutated neoantigen epitopes is a fundamental step in developing personalized cancer vaccines. This process begins with next-generation sequencing (NGS) of malignant tissues to detect tumor-specific genetic mutations (Fig. 1B). Sequencing reads are aligned to a reference genome from the patient’s normal tissue, enabling the detection of nonsynonymous mutations through mutation-calling software [83]. Detection reliability varies among SNVs, indels, and gene fusions, with SNVs exhibiting the highest sensitivity and specificity [81, 84–86]. To increase accuracy, researchers often select consensus mutations identified by at least two independent mutation-calling software programs for downstream analysis.

Another approach combines NGS with mass spectrometry to profile the HLA immunopeptidome [87–89]. Procedurally, HLA-peptide complexes are immunoprecipitated from tumor cells, and the eluted peptides are analyzed via mass spectrometry to identify HLA-bound epitopes. These analyses reveal not only the mutated neoantigens presented on HLA molecules but also their relative abundance, providing valuable insights into their immunogenic potential. Additionally, this approach enables the discovery of nonmutational antigens, such as cryptic antigens. However, sensitivity limitations and the need for large tumor samples hinder the clinical application of mass spectrometry. Despite these challenges, mass spectrometry-derived datasets are instrumental in refining bioinformatics tools that predict peptide-HLA binding affinity and antigen processing efficiency, thereby aiding in the prioritization of neoantigens for therapeutic use [90, 91].

While hundreds of nonsynonymous mutations might be identified within a tumor, only a small subset qualifies as viable neoantigens for vaccine development [82]. Selecting optimal candidates is critical to ensuring a robust immune response, minimizing antigen escape, and effectively targeting tumor cells. Several parameters are considered during this process, including binding affinity to the patient’s HLA molecules, immunogenicity, transcript expression levels, and clonality. A promising neoantigen should bind to HLA molecules and be presented on APCs or tumor cells for efficient T-cell priming and cytotoxicity. Algorithms trained on datasets derived from mass spectrometry and peptide-binding assays are now widely used to predict peptide-HLA binding affinities, filtering out candidates with poor presentation potential [91–94]. The sufficient presentation of neoantigens on tumor cells is partly determined by the abundance of neoantigen-encoding RNA transcripts, as high transcript levels correlate with increased pMHC complex density on tumor cells [95]. RNA sequencing data analysis helps quantify transcript levels, ensuring that selected neoantigens are sufficiently expressed to trigger immune responses [96, 97].

Immunogenicity, the ability of a neoantigen to provoke a strong immune response, is another key criterion. Neoantigens that highly discriminate from self-peptides are less immune tolerant and more likely to be recognized by T cells [98, 99]. Bioinformatic algorithms assess peptide similarity to the normal self-proteome, prioritizing those with the highest likelihood of immune recognition [98, 100]. Additionally, some neoantigens exhibit sequence similarity to pathogen-derived antigens, which can trigger cross-reactivity with preexisting T-cell populations primed against infectious agents [101, 102]. These neoantigens may benefit from this preformed immune memory, enhancing their therapeutic efficacy [99].

Clonality is another critical consideration in neoantigen selection. Tumors are genetically heterogeneous, with subclonal mutations arising at different stages or sites during cancer progression [103]. Clonal neoantigens, which occur early in tumorigenesis and are present in most tumor cells, are preferred over subclonal mutations. These clonal neoantigens reduce the risk of tumor escape. Mutations in genes that promote oncogenic transformation or tumor cell survival are desirable targets for vaccine development, as they are less likely to be eliminated by selective pressure [104]. Despite these advances, neoantigen selection remains challenging due to the sheer number of potential epitopes, difficulties in defining immunogenicity, and the complexity of tumor biology. Most selected mutated antigens might not be able to stimulate effective T-cell responses, and 20–30 neoantigen candidates are commonly included during the development of peptide- or mRNA-based cancer vaccines. Integrating experimental data, clinical insights, and advanced bioinformatics—particularly artificial intelligence and machine learning—will streamline the selection of neoantigen candidates. Optimized computational pipelines incorporating HLA-binding affinity, transcript expression, self-dissimilarity, and clonality will enhance the efficacy of personalized cancer vaccines. However, further technological advancements are needed to simplify and expedite individualized vaccine production for broader clinical applications.

### Cryptic antigens

Emerging evidence has shown that noncoding gene elements can encode antigen peptides presented by HLAs to induce T-cell responses. These antigens are referred to as cryptic antigens [105]. Cancer-associated epigenetic dysregulation, splicing aberrations, and cellular stress may promote the generation of tumor-specific or tumor-associated cryptic antigens, which may be immunogenic [106–108]. Human endogenous retroviral elements (HERVs) are remnants of ancient retrovirus integration events and are located throughout the human genome. These elements are usually silent in normal tissues via epigenetic modifications [109]. However, under pathological conditions such as cancer, HERVs can be reactivated, leading to the expression of viral antigens that serve as potential targets for cancer vaccination [110–114]. Circular RNAs (circRNAs) constitute another class of noncoding RNAs that play crucial roles in cancers [115]. Recent studies have revealed that circRNAs are translatable and can produce distinct peptides, making them valuable sources of TSAs. CircFAM53B is a circRNA expressed in breast cancer and melanoma that translates immunogenic peptides and elicits a T-cell response for antitumor effects [87]. Cryptic antigens are shared across multiple cancer types and are potential targets for cancer vaccines, warranting further exploration in clinical settings.

**Fig. 2:**
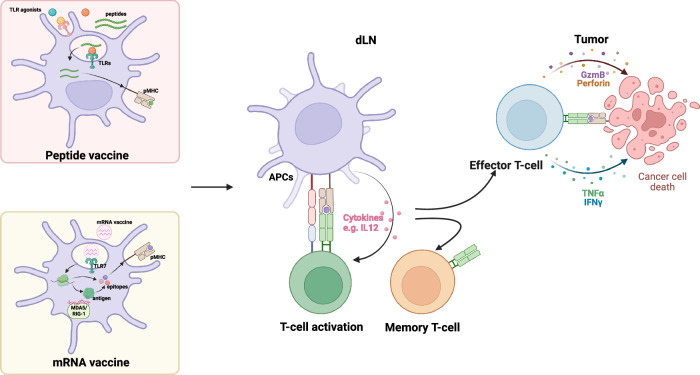
Antigen presentation of cancer vaccines. *Peptide vaccines*: Synthetic long peptides (SLPs) and adjuvants, such as Toll-like receptor (TLR) agonists, are injected to stimulate an immune response. Antigen-presenting cells (APCs) take up SLPs, process them via cross-presentation pathways, and present peptide‒MHC complexes to T cells. Adjuvants activate APCs, increasing the expression of costimulatory markers such as CD80/86 and promoting their migration to lymph nodes, where they prime and activate T cells. *mRNA* *vaccines*: mRNA vaccines encoding tumor antigens are internalized by dendritic cells (DCs) via endocytosis or macropinocytosis, followed by endosomal escape and cytosolic release of mRNA facilitated by ionizable lipids. Within the cytoplasm, mRNAs are translated into antigens, which are subsequently processed into epitopes and presented on the cell surface through interactions with MHC molecules. During this process, the mRNA vaccine might activate innate immune pathways, including TLR7, MDA5, or RIG-1, thereby stimulating innate immune responses. These antigen-presenting DCs then prime antigen-specific T cells, which differentiate into either memory T cells or effector T cells, thereby inducing tumor cell death

Overall, TSAs are promising targets for cancer vaccines. Technology advancements permit the timely design of personalized vaccines, although they are costly. Identifying appropriate antigens is the crucial first step in vaccine design. Once identified, antigens must be efficiently delivered to APCs and processed for T-cell priming in the lymphoid organ. In addition to TCR signaling, costimulatory signaling and cytokines are required for successful T-cell priming by APCs. Owing to the immune-suppressive nature of the cancer microenvironment, potent adjuvants and delivery systems are necessary to efficiently activate APCs and facilitate T-cell activation and differentiation (Fig. 2). In subsequent sections, we examine current vaccine platforms, focusing on peptide and mRNA vaccines, tracing their development through preclinical and clinical research.

## Peptide vaccine for cancer therapy

The identification of precise TCR-recognizing epitopes enables the use of synthetic peptides instead of whole tumor cell lysates or proteins in cancer vaccination [116]. Synthetic peptides encompass the minimal immunogenic regions of protein antigens, allowing for the precise modulation of immune responses. The safe and operable manufacture of neoantigen peptides ensures their clinical feasibility; however, their inherently low immunogenicity necessitates additional delivery systems and adjuvants to elicit an effective antigen-specific immune response [117]. Research on peptide-based cancer vaccines has focused on designing peptide sequences to enhance their processing and presentation by APCs [118], as well as combining them with various adjuvant systems to improve their immunogenicity [119]. Furthermore, next-generation peptide vaccines focused on developing delivery systems such as lipid-based carriers to specifically target both peptides and adjuvants to dendritic cells, thereby increasing their activation and antigen presentation for T-cell priming [120].

### Design of peptide

Initial studies identified minimal cytotoxic T-cell-recognizing epitopes, typically 8--10 amino acids in length, for vaccination purposes [117]. However, this approach results in limited antigen-specific T-cell responses and unsatisfactory clinical outcomes [121–125]. Subsequent investigations revealed that vaccination with minimal MHC-I binding epitopes could lead to T-cell anergy, possibly due to peptide binding to nonprofessional APCs, which primes T cells in the absence of costimulatory signals [126–128]. To address these challenges, synthetic long peptides (SLPs) have been proposed for vaccination. SLPs, which are usually 15--25 amino acids in length, comprise well-defined CD8 epitopes and extension sequences that may contain potential CD4 epitopes. The inclusion of CD4 epitopes enhances CD8^+^ T-cell priming, and antigen-specific CD4^+^ T cells may perform antitumor functions in the TME via various mechanisms [129–131]. Studies in mice have shown that the intracellular presentation of SLPs is more efficient than that of full-length antigens, resulting in antigen presentation to both CD4^+^ T cells and CD8^+^ T cells [132, 133].

The flanking amino acids and the linker regions between the CD4 and CD8 epitopes can significantly impact antigen presentation efficiency. One study using a DNA-based HBV vaccine in HLA-A*0201 transgenic mice revealed that the processing efficacy of an epitope strongly correlated with the presence of specific amino acid residues at the C + 1 position following the C-terminus of the CD8 epitope. The presence of an amide or positively charged amino acid residue contributes to optimal processing, whereas aromatic, negatively charged, or aliphatic residues are associated with suboptimal processing [134]. Another study utilizing the model antigen MELOE-1 screened cathepsin-sensitive linkers to join CD8 and CD4 epitopes. Cathepsin is one of the key proteases in the endosome of dendritic cells (DCs) and significantly impacts antigen processing and presentation. This research reported several candidate linkers that could enhance the cross-presentation of class I epitopes to CD8-specific T cells by up to 100-fold compared with native SLPs, with minimal impact on CD4 epitope presentation [130].

The epitope sequences that recognized by CD8^+^ T cells can be modified to enhance antigen-specific responses [117]. Heteroclitic peptides, which introduce specific residue substitutions to improve the immunogenicity of original peptides, are beneficial for tumor-associated antigens, which often exhibit poor immunogenicity due to their central tolerance [116, 135]. An important strategy for designing heteroclitic peptides is to increase the binding affinity of cytotoxic T-cell epitopes to MHC-I molecules, as this correlates with improved immunogenicity [136–138]. Simple modifications to MHC anchor residues, which substitute for suboptimal anchor residues, have proven effective in enhancing the immunogenicity of tumor-associated antigens, including gp100, p53, Trp-1, and survivin [135, 136, 139–141].

Overall, modifying the TCR-recognizing region and incorporating specific enzymatic cleavage sites can enhance the pMHC-TCR interaction and antigen presentation, thereby increasing immune responses. However, SLPs alone cannot efficiently activate T cells without providing costimulatory signals or cytokine signals. Potent immune adjuvants are required to activate APCs and provide costimulatory signals and cytokines.

### Adjuvant system

Aluminum-based adjuvants have been widely used in prophylactic vaccinations that induce an antibody response [142]. Although they are safe, aluminum salts induce an enhanced Th2 immune response with suppressed CD8^+^ T-cell activation, which has hindered their application in therapeutic cancer vaccinations [143, 144]. Thus, other adjuvants have been explored to enhance CTL responses in peptide-based cancer vaccines.

Innate immune sensors, including Toll-like receptors (TLRs), NOD-like receptors (NLRs), RIG-I-like receptors (RLRs), and cytosolic DNA sensors, detect pathogen-associated and damage-associated molecular patterns (PAMPs and DAMPs) and activate APCs to initiate adaptive immune responses [145]. Various agonists targeting these sensors have been synthesized and employed as adjuvants in peptide-based cancer vaccines [144, 146]. The structural characteristics of these immune sensors and downstream signaling pathways have been extensively characterized and reviewed elsewhere [147]. Here, we briefly describe the key immune cells and molecules that are activated by several commonly used adjuvants to enhance peptide vaccination-induced adaptive immunity.

Poly-ICLC, derived from polyinosinic–polycytidylic acid (poly I:C) and stabilized with carboxymethylcellulose and polylysine, is a commonly used adjuvant for neoantigen-based peptide vaccines [148–150]. Following endocytosis by APCs, poly-ICLC engages TLR3 on endosomal membranes and subsequently activates melanoma differentiation-associated protein 5 (MDA5) in the cytosol [151]. Preclinical studies indicate that TLR3 signaling upregulates CD80 and CD86 on DCs, enhancing interleukin-12 (IL-12) secretion and thereby facilitating T-cell priming. Concurrently, MDA5 activation induces robust type I interferon (IFN) production, which drives IL-15α/IL-15 complex formation on myeloid cells and promotes CTL expansion. Genetic ablation of the IFNα/β receptor in non–T-cell hematopoietic populations or blockade of IL-15 abrogated CTL expansion induced by poly–ICLC-adjuvanted vaccination. These findings illustrate how poly-ICLC simultaneously engages multiple innate sensors to modulate cytokine profiles and enhance CTL responses [152]. In addition to immune-stimulating activity, cationic stabilizers can induce a “proton sponge” effect that destabilizes endosomal membranes and promotes antigen cross-presentation.

Saponins are plant-derived natural products that can be formulated in liposomes with additional adjuvants, such as monophosphoryl lipid A (MPL), a TLR4 agonist. Saponin-based adjuvant systems elicit potent Th1-biased immune responses and have been approved for the development of vaccines against infectious diseases, including shingles (Shingrix) and respiratory syncytial virus (Arexvy) [153]. Upon uptake by APCs via a cholesterol-dependent mechanism, saponins destabilize endosomal membranes to facilitate antigen leakage and cross-presentation [154]. Preclinical studies have demonstrated that saponins also induce lipid body formation in CD11b^+^ DCs, promoting cross-presentation through an endosome escape-independent pathway [155, 156]. In addition to enhancing antigen uptake and cross-presentation, saponins activate the NLRP3 inflammasome and caspase-1 in lymph node-resident CD169^+^ macrophages, which recruit innate immune cells and promote DC maturation [157]. Furthermore, the aldehyde group of saponins can form Schiff base adducts with T-cell surface receptors (e.g., CD2), delivering costimulatory signals that increase Th1 cytokine production (IFN-γ and IL-2) [158].

The cytosolic DNA sensor cyclic GMP-AMP synthase (cGAS) synthesizes cyclic dinucleotides (CDNs) upon binding to cytosolic double-stranded DNA. CDNs subsequently activate STING (stimulator of interferon genes) on the endoplasmic reticulum, triggering IRF3- and NF-κB–dependent signaling pathways that drive robust production of type I IFN and other proinflammatory cytokines. The cGAS–STING axis orchestrates both innate and adaptive immune responses and is central to the antitumor effects elicited by radiotherapy and various chemotherapeutic agents [159]. Thus, STING agonists have been explored as adjuvants in cancer vaccination. In one preclinical model, the STING agonist ABM5 was covalently linked to tumor antigen peptides to facilitate delivery to the endoplasmic reticulum, thereby promoting antigen cross-presentation while simultaneously activating STING. Unlike TLR agonists, STING agonists must reach the cytosol to bind their receptor, necessitating specialized delivery systems for STING-based vaccines. The ABM5-peptide conjugate was encapsulated in lipid nanoparticles to ensure cytosolic release. This vaccine formulation was well tolerated in vivo and demonstrated significantly greater efficacy than conventional adjuvants, such as Poly-ICLC and ISCOMs [160]. In the clinic, certain patients harbor STING variants—for example, the R232H polymorphism—that exhibit reduced responsiveness to canonical CDN agonists [161]. Thus, several novel STING agonists have been developed to overcome these limitations. Li et al. reported a pH-sensitive polymer, PC7A, that activates STING by inducing STING–PC7A biomolecular condensates. PC7A binds a distinct, noncompetitive site on STING—separate from the cGAMP-binding pocket—enabling the activation of R232H STING variants [162]. When used as a vaccine adjuvant, PC7A induced potent T-cell responses [163]. Although STING agonist–based peptide vaccines have shown promise in preclinical studies, clinical exploration remains limited, and further research is needed to validate their efficacy in humans.

Current peptide-based cancer vaccines under clinical evaluation often consist of a simple admixture of TLR agonists, such as poly-ICLC, with antigenic peptides [164]. In some formulations, a water-in-oil emulsion adjuvant such as Montanide ISA 51 is included to enhance antigen retention at the injection site. However, owing to the low molecular weights of both peptides and adjuvants, these components rapidly diffuse into the systemic circulation. As a result, only a limited fraction of the antigen is internalized by local APCs. Moreover, potent adjuvants may induce systemic innate immune activation, leading to dose-limiting toxicity [165, 166]. To overcome these limitations, next-generation peptide vaccine strategies in preclinical development focus on engineering delivery platforms that codeliver both antigens and adjuvants directly to APCs, increase antigen uptake and cross-presentation, and stimulate APCs for T-cell priming while minimizing systemic exposure and toxicity.

### Toward next-generation peptide vaccination

**Fig. 3:**
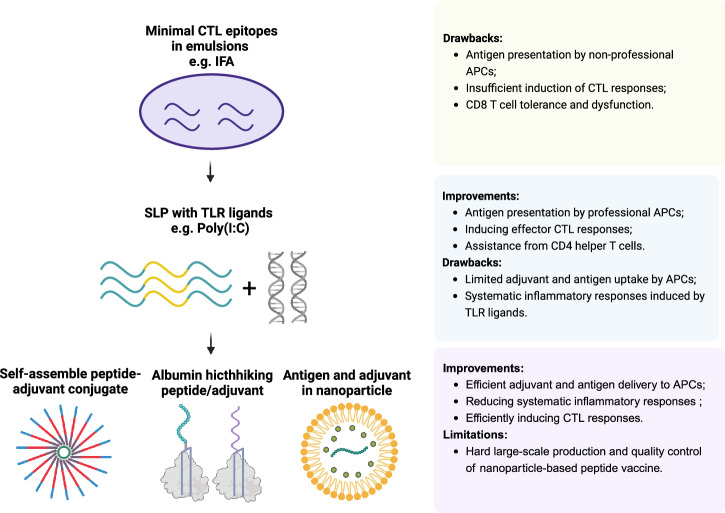
Developmental trajectory of peptide vaccines. Upper panel: Early clinical peptide vaccine formulations combining minimal CD8 ^+^ T-cell epitopes with adjuvants such as incomplete Freund’s adjuvant (IFA), creating an antigen “depot” at the injection site. However, this leads to antigen presentation by nonprofessional APCs, causing sustained chronic inflammation at the injection site. Antigen-specific T cells accumulate at the injection site and undergo tolerance and dysfunction. Middle panel: Many current clinical trials use synthetic long peptides (SLPs) mixed with Toll-like receptor (TLR) agonists, such as polyICLC. SLPs promote antigen presentation by professional APCs and may contain CD4 ^+^ T-cell epitopes, which help recruit CD4^ +^ T cells to assist in CD8 ^+^ T-cell priming. This improves T-cell priming and effector T-cell generation. However, owing to the small size of TLR agonists and SLPs, most of the injected reagents leak into the circulation, with limited antigen and adjuvant uptake by APCs. Additionally, some TLR agonists can induce systemic proinflammatory responses, resulting in toxicity. Lower panel: New-generation peptide vaccines use particulate delivery systems for antigens and adjuvants, which improve lymphoid tissue targeting. Antigens and adjuvants can be designed to self-assemble into nanometer-scale complexes or be incorporated into lipid-based nanoparticles, which are preferentially taken up by APCs. This design allows for the efficient codelivery of antigens and adjuvants, reducing systemic inflammatory responses and inducing robust T-cell responses. However, large-scale production and quality control of lipid nanoparticle-based vaccines remain significant challenges. Another strategy involves conjugating peptides or adjuvants to moieties that bind albumin, which increases APC uptake and enhances T-cell responses

Various delivery platforms, including PLGA nanoparticles, hydrogels, inorganic nanomaterials, and liposomes, have been developed to increase the efficacy of peptide-based cancer vaccines. Among these, lipid-based carriers have received the most extensive investigation because of their favorable biocompatibility, structural versatility, and ease of functionalization. In the following section, we focus on lipid-based platforms and discuss the diverse strategies employed to optimize lipid carrier design for the efficient codelivery of antigens and adjuvants to APCs (Fig. 3).

#### Physicochemical properties influencing lymph node drainage and cellular uptake

The physicochemical properties of lipid nanoparticles—including size, surface charge, hydrophobicity, and flexibility—play critical roles in determining their lymphatic drainage and cellular uptake [167]. Particle size is a key determinant of biodistribution: nanoparticles smaller than 10 nm in diameter are prone to rapid diffuse into the bloodstream, whereas those larger than 100 nm are often retained at the injection site. Intermediate-sized particles are more likely to enter lymphatic vessels and reach draining lymph nodes [168]. Smaller particles are generally internalized by immune cells through multiple endocytic pathways, including clathrin- and caveolae-mediated endocytosis, which are efficient routes for antigen processing. In contrast, larger particles are primarily taken up by phagocytosis, a process typically restricted to professional phagocytes and associated with lower cross-presentation efficiency [169]. Surface charge and hydrophobicity also influence biodistribution and cellular uptake in opposing ways. Positively charged or hydrophobic particles are more likely to interact with and become entrapped in the negatively charged interstitial matrix, potentially limiting lymphatic drainage. However, such particles more readily associate with the negatively charged plasma membrane, facilitating endocytosis and enhancing endosomal escape. Conversely, hydrophilic or negatively charged particles exhibit improved lymphatic transport but may be less efficiently internalized by APCs [167, 170]. Flexibility represents an additional, independent factor influencing lymph node trafficking. In one study, lipid nanodiscs exhibited greater endothelial penetration and lymphatic drainage than liposomes did, which was attributed to their greater deformability [171]. However, the effect of particle elasticity on cellular uptake remains controversial. For example, Palomba et al. demonstrated that rigid nanoparticles were internalized five times more efficiently than their soft counterparts were internalized, whereas another study reported that liposomes with higher membrane fluidity were more readily taken up by APCs [50, 172]. These conflicting findings may reflect differences in nanoparticle composition and experimental context, suggesting that the role of particle elasticity in immune cell uptake is likely material- and system dependent, warranting further investigation.

#### Lipid component selection for enhanced delivery efficiency

The lipid composition of nanoparticles plays a pivotal role in determining their physicochemical properties, biodistribution, and immunological performance. The chemical structure of the lipid headgroup, in particular, modulates surface charge and immunomodulatory characteristics [173]. Cationic lipids confer a positive surface charge, which can enhance cellular uptake but may also induce undesired inflammatory responses through activation of the complement system and myeloid cells [174, 175]. Additionally, lipid headgroups can interact electrostatically or hydrophobically with serum proteins, leading to the formation of a protein corona that significantly influences nanoparticle fate in vivo, often enhancing clearance by the reticuloendothelial system [176, 177]. To mitigate nonspecific protein adsorption, polyethylene glycol (PEG)-conjugated lipids are frequently incorporated into lipid formulations. While PEGylation can reduce protein corona formation and prolong the circulation time, a high density of PEG chains may hinder their cellular uptake and impair endosomal escape [178]. Alternatively, tailoring the lipid headgroup to favor interactions with specific serum proteins can promote selective delivery to target tissues via receptor-mediated mechanisms [50, 179]. For example, precoating lipid particles with targeting moieties such as anti-CD40 antibodies has emerged as a strategy to direct particles toward antigen-presenting cells (APCs) and enhance their in vivo bioactivity [177]. The lipid tail also critically influences nanoparticle behavior. Unsaturated, short, or branched lipid tails increase membrane fluidity, facilitating faster antigen release and endosomal escape, but may compromise thermal stability [180–182]. In a systematic study evaluating the effects of both lipid headgroups and tails in a liposome-based peptide vaccine platform, phosphoethanolamine headgroups were found to preferentially bind serum complement protein C3, mediating spleen-targeted delivery in a C3-dependent manner. Additionally, lipids bearing 1,2-dioleoyl-sn-glycero tails demonstrated enhanced fluidity and a favorable phase transition temperature, leading to improved cellular uptake and robust T-cell activation [50]. In addition to the structural lipids that form the lipid bilayer, functional excipients such as PEGylated lipids and cholesterol are commonly incorporated to increase particle stability and circulation time [180]. Moreover, lipids can be chemically modified to allow covalent conjugation with key functional molecules such as antigenic peptides or immune-stimulatory adjuvants, further expanding their utility in rational vaccine design.

#### Incorporating antigens and adjuvants into lipid carriers

Antigenic peptides and adjuvants can be integrated into lipid-based delivery systems either by noncovalent or covalent attachment to the lipid surface or by encapsulation within the lipid core. Additionally, small hydrophobic adjuvants can be embedded within the lipid bilayers of liposomes [183]. Compared with noncovalent attachment or encapsulation, covalent conjugation allows for precise control over the surface density of antigens or adjuvants by modulating the chemical linkers incorporated into the lipid structure. Covalent linkages also increase the molecular loading efficiency and improve the consistency and stability of lipid particles [184]. Preclinical studies have demonstrated that lipid carriers covalently conjugated with peptides and adjuvants exhibit potent immunogenicity. For example, Kuai et al. successfully conjugated antigens and CpG oligonucleotides to high-density lipoprotein (HDL)-mimicking nanodiscs, markedly enhancing codelivery to lymphoid organs and sustaining antigen presentation by dendritic cells. This strategy resulted in a 47-fold increase in neoantigen-specific cytotoxic T lymphocytes (CTLs) compared with soluble vaccines and a 31-fold increase compared with the level of CpG in Montanide, one of the most potent adjuvants currently in clinical use [185]. Another study investigated the effects of different antigen–adjuvant conjugation strategies on the immunogenicity of spherical nucleic acid (SNA)-based liposome vaccines. In this study, CpG oligonucleotides were anchored on the liposome surface, while antigens were either (i) encapsulated in the liposome core (E structure), (ii) covalently conjugated to surface-anchored oligonucleotides (A structure), or (iii) hybridized to CpG through complementary oligonucleotides chemically linked to the antigen (H structure). Among these, the H structure enables the most efficient codelivery of antigen and adjuvant in vivo and best synchronized antigen presentation with costimulatory molecule expression, thereby eliciting superior T-cell responses [186]. Furthermore, the chemical linkers used for covalent conjugation can be engineered to allow either rapid or controlled release of antigens in response to the acidic and redox conditions of endosomes. In one study, antigens were conjugated via either noncleavable or redox-responsive cleavable linkers, and faster-releasing linkers led to stronger immunostimulatory effects [187]. Collectively, these findings highlight that the method of incorporating antigens and adjuvants into lipid particles, along with the design of chemical linkers and the structural configuration of the vaccine, plays a crucial role in determining vaccination efficacy.

#### Identifying optimal lipid particle formulations by screening

Due to the numerous technical variables influencing the efficacy of lipid particle–based peptide vaccines, rational and systematic large-scale screening may be necessary to identify optimal formulations. While many studies have focused on optimizing the lipid composition of lipid nanoparticles to increase mRNA delivery and translation, relatively few have systematically evaluated the key factors that govern the immunogenicity of peptide-loaded lipid particles [188, 189]. Notably, Gokay et al. employed high-throughput screening combined with machine learning to investigate the design of spherical nucleic acid (SNA)-based peptide vaccines. They evaluated 11 design parameters—including the core diameter, lipid composition, conjugation chemistry, and density of antigens and adjuvants—by generating hundreds of structurally distinct SNAs. A mass spectrometry–based assay was used to assess immune activation rapidly, and machine learning models were applied to quantitatively predict immunostimulatory effects. Through this approach, they identified and ranked critical factors influencing vaccine efficacy and developed a nonlinear model for predicting the immunogenic potential of SNAs [190]. Despite this progress, further efforts are needed to systematically define generalizable design principles for lipid particle–based peptide vaccines.

#### Barriers to the clinical application of lipid carrier-based peptide vaccination

Despite remarkable success in preclinical studies, significant challenges remain in synthesizing and scaling up clinical-grade complex formulations containing multiple components, hindering the application of diverse vaccine nanoparticles in clinical trials. Additionally, the instability of such formulations—both physically and chemically—limits long-term storage and clinical applicability [191, 192]. Current neoantigen-based vaccination strategies further complicate the clinical translation of lipid particle–based peptide vaccines. While preclinical studies often employ well-defined model antigens, such as the MHC-I epitope of ovalbumin, the HPV16 E7 protein, or the ADPGK neoantigen in MC38 cells, human neoantigen vaccination requires the synthesis of multiple, patient-specific peptides. These neoantigens often differ in their chemical properties, leading to inconsistent encapsulation efficiencies in lipid particles. Moreover, when conjugated to lipid particle surfaces, these peptides can significantly alter surface characteristics such as hydrophobicity and charge, resulting in final products with varying stability and physicochemical profiles. These factors pose substantial challenges for the clinical application of lipid particle–based neoantigen peptide vaccines.

Another strategy to improve lymph node trafficking and APC uptake is “albumin hitchhiking,” where adjuvants and peptides are linked to albumin-binding lipids, promoting lymphatic drainage and efficient APC targeting in vivo [193–198]. For example, Zhu et al. developed self-assembling albumin-vaccine nanocomplexes (AlbiVax) by conjugating molecular vaccines with Evans blue, a dye with a high affinity for albumin. Compared with the benchmark IFA adjuvant, AlbiVax achieved 100-fold more efficient codelivery of CpG and antigens to lymph nodes, eliciting ten-fold higher frequencies of peripheral antigen-specific CD8^+^ T cells with immune memory [193]. Another study utilized a similar albumin hitchhiking strategy by tagging peptides and adjuvants via a PEG linker to amphiphilic lipid tails that bind to albumin in vivo. This amphiphilic peptide vaccine also accumulated in lymph nodes and enhanced the generation of antigen-specific CD8 ^+^ T cells [194, 198]. These designs enhance vaccine delivery efficiency through simple molecular modifications, enabling stable, large-scale production suitable for clinical application.

In summary, next-generation peptide vaccines aim to codeliver antigens and adjuvants to the APCs of draining lymph tissues while minimizing peripheral toxicity via various delivery systems. While lipid–nanoparticle formulations have exhibited superior efficacy in preclinical studies, their clinical application is hindered by complex manufacturing procedures and challenges in large-scale, consistent production. Other strategies, such as albumin hitchhiking, may be clinically practicable; however, further investigations are needed on reliable delivery systems for clinical application.

### Clinical trials

Peptide vaccines have demonstrated good safety profiles in clinical trials. However, several large phase III cancer vaccine studies in patients with advanced disease have reported limited survival benefits [121–125]. The failure of these early clinical trials can be attributed to several factors, including the use of tolerant TAA epitopes, ineffective adjuvants, insufficient antigen delivery, and a short half-life inside dLN [199].

Some ongoing clinical studies using neoantigen peptide vaccines have shown promising preliminary results (Table 1). One study tested a poly-ICLC adjuvant neoantigen peptide vaccine in glioblastoma patients following surgery and radiotherapy. In patients who do not receive dexamethasone, vaccination induces the generation of a memory immunity with polyfunctional neoantigen-specific CD4^+^ and CD8^+^ T cells in circulation. Single-cell TCR analysis revealed that TCRαβ clonotypes were shared among tumor-specific T cells from tumor tissue and peripheral blood, indicating that T cells migrated from the peripheral blood to the tumor. Intratumoral T cells upregulate multiple inhibitory molecules, providing a rationale for combining this therapy with ICB [148].

**Table 1:** Selected clinical trials of therapeutic DC vaccine, peptide vaccine, mRNA vaccine

Clinical trial (phase)	Target antigens	Platform	Adjuvant	Indication	Combination therapy	Injection	Responses	Major irAEs
PEPTIDE VACCINE								
NCT00003638;NCT00046371 (Phase 3) [364]	Sialyl-Tn	Theratope	KLH	MBC	Cyclophosphamide	S.C.	Median OS: 23.1 months vs 22.3 months (P = 0.916)	Well tolerated
NCT01015443 (Phase 3) [123]	MUC1	Tecemotide (L-BLP25)	Liposomal monophosphoryl lipid A	Unresectable stage III NSCLC	Cyclophosphamide	S.C.	Median OS: 25.6 months vs 22.3 months (HR 0.88; P = 0.123)	Well tolerated
NCT00480025 (Phase 3) [121]	MAGE-A3	GSK1572932A	Liposomal AS15	Completely resected stage IB–II NSCLC	/	I.M.	Fail to meet primary end points of extended DFS	Treatment did not increase AEs compare to placebo
NCT00019682 (Phase 3) [15]	gp100	/	IL2 plus Montanide™ ISA51	Stage III or stage IV melanoma	High dose IL2	S.C.	OS: 17.8 months vs 11.1 months (P = 0.06) PFS: 2.2 months vs 1.6 months (P = 0.08)	Toxicity due high dose IL2 treatment
NCT01480479 (Phase 3) [365]	EGFRvIII	CDX-110	KLH and GM-CSF	GBM	Temozolomide	I.D.	No survival benefit	Not toxicity increment due to vaccination
NCT00796445 (Phase 3) [40]	MAGE-A3	GSK 2132231 A	AS15	Resected melanoma	/	I.M.	Failed to meet primary end point of DFS	Severe AEs: 14% in the MAGE-A3 group; 12% in the placebo group
NCT00358566; NCT00425360 (Phase 3) [366]	Telomerase	GV1001	GM-CSF	Locally advanced and/or metastatic pancreatic cancer	Chemotherapy	I.D.	No OS difference	Equal AEs between treatment and control group
NCT02854072 (Phase3) [363]	Telomerase	GV1001	GM-CSF	Advanced PDAC having high serum eotaxin	Chemotherapy	I.D.	Improved OS and TTP	Equal AEs between treatment and control group
UMIN000016954 (Phase3) [367]	Five cancer-testis antigens	S-588410	Montanide™ ISA 51 VG	ESCC	/	S.C.	Failed to reach the primary endpoint	Injection site reactions
NCT05232916 (Phase 3) [368]	HER2	GLSI-100	GM-CSF	HER2/neu positive breast cancers	/	I.D.	On going	Well tolerated
NCT02128126 (phase 1/2) [369]	HPV16 E6 E7	/	ISA101	Advanced HPV16+ cervical cancer	Pegylated IFNα and chemotherapy	S.C.	Tumor regressions were observed in 43% of 72 evaluable pts	Chemotherapy induced TRAEs
NL21215.000.08 (phase 1/2 study) [370]	HPV16 E6 E7	/	ISA101	HPV16-induced high-grade vulvar and vaginal intraepithelial neoplasia	imiquimod	S.C.	Vaccine-induced clinical responses were observed in 18 of 34 (53%) pts	Well tolerated
NCT02821494 (phase I) [371]	HPV16 E6	Amplivant-SLP	Amplivant	HPV16+ oropharyngeal cancer	/	I.D.	On going	Tolerated
NCT04853017 (Phase1) [198]	mKRAS	Amphiphile -SLP	Amph-CpG-7909	Kras mutated tumors	/	S.C.	mKRAS-specific T-cell responses and tumor biomarker responses were observed in 21 of 25 pts	Well tolerated
NCT03047928 (Phase 1/2) [25]	PDL1/IDO	/	Montande	Metastatic melanoma	Nivolumab	S.C.	80% ORR, 43% complete responses26 months PFS	Four pts (13%) experienced grade 3–4 AEs
NCT05269381 (Phase 1/2) [372]	Neoantigens	IO102/IO103	GM-CSF	Solid tumors	Pembrolizumab/cyclophosphamide	S.C.	On going	Well tolerated
NCT02897765 (Phase 1b) [149]	Neoantigens	NEO-PV-01	Poly-ICLC	Melanoma, Lung Cancer or Bladder Cancer	Nivolumab	S.C.	De novo neoantigen-specific CD4+ and CD8 + T-cell responses observed	Well tolerated
NCT02287428 (Phase 1b) [148]	Neoantigens	/	Poly-ICLC	Newly Diagnosed GBM	RT Plus Pembrolizumab	S.C.	Neoantigen-specific CD4+ and CD8 + T-cell responses observed	Well tolerated
NCT01970358 (Phase1) [150]	Neoantigens	NeoVax	Poly-ICLC	Melanoma	/	S.C.	Evidence of tumor infiltration by neoantigen-specific T-cell after vaccination and epitope spreading	Well tolerated
NCT03633110 (Phase 1) [373]	Neoantigens	GEN-009	Poly-ICLC	Solid tumors	aPD-1	S.C.	Evidence of tumor infiltration by neoantigen-specific T-cell after vaccination and epitope spreading	Well tolerated
MRNA VACCINE								
NCT05933577 (mRNA-4157, phase 3) [288]	Neoantigens	LNP	/	Resected high-risk stage II-IV melanoma	Pembrolizumab	I.M.	On going	Well tolerated
NCT06077760 (mRNA-4157, phase 3) [289]	Neoantigens	LNP	/	Resected and chemotherapy-treated stage II–IIIB (N2) NSCLC	Pembrolizumab	I.M.	On going	Well tolerated
NCT05141721 (GRANITE, phase 2/3) [304]	Neoantigens	LNP	/	MSS-CRC	ChAd68, fluoropyrimidine/bevacizumab	I.M.	Preliminary data from the phase 2 portion: PD was observed in 11% vs 38% in GRANITE vs the control. An early trend in PFS data was seen with GRANITE	Most of the AEs observed were grade 1 or 2
NCT04526899 (BNT111, phase 2) [37]	NY-ESO-1, MAGE-A3, tyrosinase, and TPTE	LPX	/	Unresectable stage III or IV melanoma	Cemiplimab	I.V.	A statistically significant improvement in ORR in patients treated with BNT111 in combination with aPD-1 compared to historical control	Well tolerated
NCT05968326 (BNT122, phase 2)	Neoantigens	LPX	/	Surgically resected pancreatic cancer	Atezolizumab and mFOLFIRINOX	I.V.	On going	Well tolerated
NCT04486378 (BNT122-01, phase 2) [374]	Neoantigens	LPX	/	Patients with resection Stage II (high risk)/Stage III CRC who are ctDNA positive	/	I.V.	All 11 pts were disease-free at median follow-up of 640 days. Autogene cevumeran induced de novo T-cell response against >1 antigen in 11/11 assessed pts; Neoantigen-specific T cells had predominantly effector memory phenotype and expressed PD-1	Well tolerated
NCT04534205 (BNT113, phase 2) [375]	HPV16 E6 and E7	LPX	/	Unresectable recurrent or metastatic HPV16 + PD-L1 + HNSCC	Pembrolizumab	I.V.	27% (4/15) pts experienced CR, 13% (2/15) PR. PFS was 3.9 months, OS was 22.6 months. 2/3 pts assessed showed de novo vaccine-induced T-cell responses against E6 and E7 oncoproteins	Grade ≥3 AEs related to BNT113 and pembrolizumab occurred in 2 pts each
NCT05557591 (BNT116, phase 2)	MAGE A3, CLDN6, KK-LC-1, PRAME, MAGE A4, MAGE C1	LPX	/	NSCLC	Cemiplimab	I.V.	On going	On going
NCT05533697 (mRNA-4359, phase 1/2) [376]	PD-L1, IDO1	LNP	/	Advanced/refractory solid tumors	/	I.M.	8/16 (50%) response-evaluable pts had SD. Either PD-L1 or IDO1 T-cell responses were detectable in 13/14 (93%) pts	AEs grade 1–2; a maximum tolerated dose was not reached.
NCT03953235 (SLATEv1, phase 1/2) [305]	20 shared neoantigens targeting oncogenes (KRAS and TP53)	LNP	/	Advanced metastatic solid tumors	ChAd68, aPD-1, aCTLA-4	I.M.	ORR was 0%, and median PFS and OS were 1.9 months and 7.9 months	5% had grade 3/4 AEs
NCT04503278 (BNT211-01, phase 1/2) [308]	CLDN6	LPX	/	Relapsed/refractory CLDN6+ solid tumors	CLDN6-CAR T	I.V.	ORR was 38% (20/52); DCR was 69% (36/52)	Grade ≥ 3 TRAEs in 64% pts; serious related AEs in 39% pts
NCT04573140 (phase 1/2) [244]	pp65	LPA	/	Primary MGMT unmethylated GBM	/	I.V.	On going	On going
NCT01817738 (CV9104, phase1/2) [295]	PSA, PSMA, PSCA, STEAP1, PAP, and MUC1	Protamine	/	mCRPC	/	I.D.	No significant improvement in PFS and OS	51.1% had grade ≥ 3 AEs
NCT01915524 (CV9202, phase 1) [297]	NY-ESO-1, MAGE-C1, MAGE-C2, survivin, 5T4, MUC1	Protamine	/	Stage IV NSCLC	Local irradiation (with or without pemetrexed and EGFR tyrosine-kinase inhibitor)	I.D.	One patient had a PR in combination with pemetrexed maintenance, and 46.2% achieved SD	15.4% had grade ≥ 3 AEs
NCT05938387 (CVGBM, phase 1) [377]	Eight segments derived from four GBM-relevant TAAs	LNP	/	Newly diagnosed and surgically resected MGMT-unmethylated GBM	/	I.M.	On going	6% had grade ≥ 3 AEs
NCT03394937 (E011-MEL, phase 1) [378]	Tyrosinase, gp100, MAGE-A3, MAGE-C2, PRAME	/	mRNA encoding CD40L/CD70/caTLR4	Resected stage IIc/III/IV cutaneous melanoma	/	I.N.	Vaccine-induced immune responses were detected in 7/19 pts	No serious TRAEs Grade 3 or higher were reported

*pts* patients, *OS* Overall survival, *PFS* Progress-free survival, *RFS* Recurrence-free survival, *SD* Stable disease, *DFS* Disease-free survival, *ORR* Overall response rate, *PD* Progressive disease, *CR* Complete response, *DCR* Disease control rate, *TTP* Time to progress, *irAEs* Immune-related adverse events, *TRAEs* Treatment emergent adverse events, *I.V*. Intravenous injection, *I.D*. Intradermal injection, *S.C*. Subcutaneous injection, *I.M*. Intramuscular injection, *I.N*. Intranodal injection, *PAP* Prostatic acid phosphatase, *pp65* Phosphoprotein 65, *gp100* Glycoprotein 100, *MAGE* Melanoma-associated antigen, *NY-ESO-1* New York esophageal squamous cell carcinoma-1, *Sialyl-Tn* Sialyl-Thomsen-nouveau, *MUC1* Mucin 1, *EGFRvIII* Endothelial growth factor receptors, *HER2* Human epidermal growth factor receptor 2, *HPV16* Human papillomavirus type 16, *mKRAS* Mutant Kirsten rat sarcoma gene, *PDL1* Programmed cell death 1 ligand 1, *IDO1* Indoleamine2,3-dioxygenase-1, *TPTE* Transmembrane phosphatase with tensin homology, *CLDN6* Claudin

Another study combined a poly-ICLC adjuvant neoantigen peptide vaccine with nivolumab in patients with advanced melanoma, non-small cell lung cancer, or bladder cancer. This combination therapy induced durable neoantigen-specific T cells with cytotoxic potential, which migrated and infiltrated into tumor tissues. Importantly, vaccination also helps induce T-cell responses against nonvaccinated neoantigen epitopes, which may prevent epitope escape and tumor relapse. Notably, patients who experienced this epitope spreading response had longer progression-free survival (PFS) than those who did not [149]. Similar findings were reported in melanoma patients vaccinated with neoantigens, where memory T-cell formation and epitope spreading were observed [150].

For shared tumor antigens, novel vaccine formulations have yielded promising clinical results. For example, a synthetic TLR2 ligand was conjugated to SLPs derived from the HPV16 virus. This vaccination approach effectively induced robust HPV16-specific T-cell immunity without eliciting severe side effects in patients with HPV16-positive (pre)malignancies [200]. Another study conjugated mutant KRAS (mKRAS) peptides and a CpG adjuvant to albumin-binding lipids via a PEG linker. This vaccine was well tolerated in vivo, with mKRAS-specific T-cell responses observed in 21 of the 25 patients and tumor biomarker responses (longitudinal ctDNA or tumor antigen) noted in 21 of the 25 patients, including biomarker clearance in six of the patients. T-cell responses are correlated with PFS; patients with T-cell responses above the median exhibit delayed cancer recurrence compared with those with responses below the median [198].

These studies suggest that the clinical success of peptide vaccines may depend on several factors: the use of multiple neoantigen epitopes with potent adjuvants to overcome immune tolerance and epitope loss, the combination of vaccination with immune modulators such as immune checkpoint blocking antibodies to mitigate immune suppression, and the application of novel vaccine formulations that efficiently target antigens and adjuvants to APCs. Whether and how vaccines can be combined with first-line therapies, such as radiation and chemotherapy, remain to be explored.

### mRNA vaccine

DNA-based vaccination was widely explored before the breakthrough of mRNA-based vaccination. However, complex electroporation procedures for delivering DNA into the nucleus for transcription and translation are needed, but show low efficiency and introduce the risk of genomic integration of transfected DNA molecules. The majority of DNA molecules are taken up by muscle cells but not APCs, leading to insufficient antigen presentation in the dLN [201]. The mRNA vaccine delivers mRNA molecules into the cytosol, allowing for high quantities of antigen production through translation and preventing the risk of genomic integration. It is relatively easy to include multiple antigen-encoding minigenes in the designed constructs. Thus, mRNA vaccination is currently more intensively explored [202].

The concept of mRNA-based therapeutics dates back to 1984, when the first biologically active mRNA was synthesized to express proteins in vitro [203]. By the early 1990s, researchers demonstrated that naked or liposome-encapsulated mRNAs could induce protein expression and activate antigen-specific cytotoxic T cells in vivo [204, 205]. These findings establish mRNA as a promising therapeutic strategy because of its low risk of genome integration, ease of design and modification, ability to encode multiple antigens, and rapid antigen production. In 1995, the first cancer mRNA vaccine encoding carcinoembryonic antigen (CEA) successfully induced anti-CEA antibodies in mice [206]. However, the development of mRNA-based cancer vaccines has been slower than that of other cancer vaccine platforms because of multiple challenges such as mRNA instability, inefficient delivery methods, and high intrinsic immunogenicity. Notably, the cornerstone discovery that nucleoside modifications reduce immunogenicity while enhancing protein production was instrumental in the successful application of mRNA vaccines against COVID-19 [207–209]. These findings have inspired the development of mRNA-based cancer vaccines. Current studies focus on optimizing the mRNA structure to improve stability and enhance antigen expression levels or duration, developing efficient delivery systems to increase transfection efficiency, targeting mRNA delivery to specific tissues or cells, modulating the adjuvant effects of mRNA vaccines to elicit stronger adaptive immune responses, and combining mRNA vaccines with other immunotherapies to achieve superior tumor control.

### In vitro transcribed mRNA

The in vitro transcribed (IVT) mRNA comprises a 5ʹ cap, a 5ʹ untranslated region (UTR), an open reading frame (ORF) that encodes the antigen, a 3ʹ UTR, and a poly(A) tail. Optimizing these structural elements can increase mRNA stability and improve translation efficiency [210, 211]. High-throughput screening technologies, combined with machine learning algorithms, are employed to identify optimal 5ʹ and 3ʹ UTR sequences from UTR libraries [212–215]. Furthermore, coding sequence (CDS) optimization is achieved through codon optimization, GC content adjustment, and secondary structure improvement via computer algorithms [216–219]. Maintaining the length of the poly(A) tail within 120–150 nucleotides and incorporating short sequences or thiophosphates into poly(A) sequences have been shown to protect mRNAs from deacetylation and degradation [202, 211, 220–223].

Linear mRNA is rapidly degraded following translation, which limits the production of sufficient antigen for triggering immune responses. Various RNA types have been explored to increase the stability and duration of RNA. Commonly investigated RNA molecules include circular RNA (circRNA), self-amplifying RNA (saRNA), and trans-amplifying RNA (taRNA). CircRNAs exhibit increased stability due to the absence of free ends, which prevents recognition and degradation by nucleic acid exonucleases [224–228]. SaRNAs contain a sequence encoding RNA-dependent RNA polymerase (RDRP), which enables their self-amplification. This design extends protein expression while reducing the required mRNA vaccine dose [49, 229]. TaRNA is a modified form of saRNA generated by splitting saRNA into two distinct RNA molecules, one encoding the replicase sequence and the other encoding the protein of interest, which is amplified by the replicase to prolong antigen expression [202, 230]. Several of these RNA designs have been evaluated in preclinical cancer vaccine studies and have demonstrated potent antitumor immunity [49, 227, 228].

### Current mRNA vaccination formulations in the clinic

**Fig. 4:**
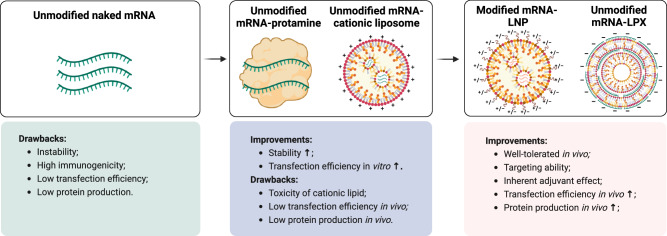
Developmental trajectory of cancer mRNA vaccines. Early clinical trials of mRNA vaccines focused on the direct injection of unmodified, naked mRNA via intranodal administration. However, this approach faces many challenges, including the instability and high immunogenicity of mRNAs, low transfection efficiency into antigen-presenting cells (APCs), and low protein production. Therefore, some formulated vectors, such as cationic protamine and liposomes, have been developed to encapsulate unmodified mRNAs. While these formulations improve mRNA stability and transfection efficiency in vitro, their in vivo efficacy remains constrained by low transfection efficiency and protein production and the toxicity associated with cationic components. More recently, LNP- or LPX-encapsulated mRNA vaccines have been frequently utilized, along with modified (m1ψ) mRNAs, which exhibit reduced immunogenicity. Thus, the transfection efficiency and protein production are enhanced in vivo. These mRNA vaccines are generally well tolerated and possess inherent adjuvant effects that stimulate innate immune responses, triggering antigen-specific T-cell activation. Moreover, certain delivery systems, such as spleen-targeting mRNA-LPX, can deliver mRNAs more specifically to the spleen

Naked mRNA is rapidly degraded after administration because of the ubiquitous presence of ribonucleases (RNases). Additionally, its negative charge and hydrophilic nature limit its cellular uptake and result in inefficient endosomal escape, thereby restricting its therapeutic application [231]. Therefore, delivery systems that protect mRNAs from degradation, facilitate their uptake by APCs, and enable them to escape from endosomes for cytosolic protein translation are crucial for the effective delivery of mRNA vaccines (Fig. 4) [232].

#### mRNA‒lipid nanoparticles (LNPs)

LNPs are the preferred delivery system for mRNAs because of their proven success in COVID-19 mRNA vaccines, such as mRNA-1273 and BNT162b2 [233, 234]. LNPs typically consist of four lipid components: an ionizable lipid, cholesterol, a phospholipid, and a PEGylated lipid. LNPs encapsulate mRNA payloads via electrostatic interactions, forming uniform spheres with diameters ranging from 50 to 150 nm. The ionizable lipid, which carries a positively charged head group at low pH, facilitates the encapsulation of negatively charged nucleic acid payloads. The head group becomes uncharged at physiological pH, which reduces toxicity [235]. Following cellular uptake, the ionizable lipid is protonated within acidic endosomes, where it interacts with anionic endosomal phospholipids to disrupt the membrane, thereby enabling endosomal escape and cytosolic release of the payloads [236]. The clinically approved ionizable lipids include DLin-MC3-DMA (MC3), SM-102, and ALC-0315, which have been used in patisiran, mRNA-1273, and BNT162b2, respectively [237]. Cholesterol enhances the structural stability of LNPs and promotes membrane fusion. Phospholipids such as DOPE, DSPC, and DOPG act as helper lipids, enhancing structural stability and promoting endosomal escape [232]. PEGylated lipids, such as PEG-DMG and PEG-DSPE, are hydrophilic and assemble on the LNP surface. These lipids reduce nanoparticle aggregation and prolong their circulation time by reducing clearance by the mononuclear phagocytic system [238]. Additionally, functionalized PEGylated lipids can modify the LNP surface, allowing for conjugation with specific ligands or molecules for targeted delivery to specific cells or tissues. While PEGylation helps prolong circulation time, it may reduce LNP cellular uptake by limiting serum protein adsorption, membrane fusion, or endosome escape [178, 239, 240].

#### RNA-lipoplex (RNA-LPX)

RNA-LPX, developed by BioNTech, is a widely used lipoplex for delivering mRNA vaccines against cancer via intravenous administration. RNA-LPX is formed through the self-assembly of RNA with dedicated cationic liposomes, resulting in a topological transformation from liposomes into compact nanoparticles with diameters of 200–400 nm. Notably, RNA-LPX mainly targets RNA to the spleen because of the charge ratio between the cationic lipid and the RNA. The negative charge caused by excess RNA results in the accumulation of RNA-LPX in the spleen [241]. Following intravenous (i.v.) administration, splenic macrophages exhibit the highest uptake of RNA-LPX within one hour, whereas conventional dendritic cells (cDCs) demonstrate the greatest efficiency in protein translation [242]. The primary mechanism for RNA-LPX internalization by immature DCs is macropinocytosis rather than receptor-mediated endocytosis or phagocytosis [242].

#### Other delivery systems

Several other delivery platforms have been developed to deliver mRNA vaccines in vivo. Protamine–mRNA complexes utilize cationic, arginine-rich protamine peptides to condense and deliver mRNA. Lipopolyplex (LPP) consists of a polymeric polyplex mRNA core encapsulated within a phospholipid bilayer shell. Like RNA-LPX, LPP enters DCs through macropinocytosis [243]. Recently, “onion-like” multilamellar RNA lipid particle aggregates (LPAs) with a diameter of 200–500 nm have been developed. Through novel packaging strategies, LPAs enhance payload packaging and immunogenicity [244]. Following i.v. injection, LPAs are entrapped in reticuloendothelial organs such as the spleen, lymph nodes, liver, lungs, and kidneys. Unlike other platforms that deliver mRNA to APCs, LPAs transfect fibroblastic reticular cells (FRCs) and may provide antigens to APCs via locoregional release and direct contact.

In conclusion, the diameter, charge, and molecular composition of nanoparticles are critical factors in determining their tissue distribution and cellular targeting, although the underlying mechanisms remain unclear. Further surface modifications of nanoparticles with targeting moieties, such as antibodies or small-molecule ligands, can achieve active targeting to specific cell types [236]. However, as various lipid components and delivery carriers are used in RNA vaccination, their pharmacodynamic properties and in vivo metabolism need to be thoroughly evaluated. Clinical procedures for assessing the efficacy and safety of these carriers should also be established to guide the development of clinically viable RNA carriers.

### Adjuvant effect

Adjuvants are key components of vaccines that increase the magnitude, breadth, and durability of the immune response [245]. Both lipid nanoparticles and mRNAs may function as effective adjuvants, stimulating innate immune responses that support the priming and maintenance of adaptive immunity. This built-in adjuvant effect elicits robust antitumor activity, obviating the need for additional adjuvants [246].

#### Adjuvant effect of mRNAs

Synthetic mRNAs can activate innate immune sensors, including Toll-like receptors (TLR3, TLR7, and TLR8), leading to the production of proinflammatory cytokines [207]. Additionally, double-stranded RNA (dsRNA), a byproduct generated during in vitro transcription, can stimulate various innate immune pathways via sensors such as TLR3, RIG-I, and MDA5 [245, 246]. Induced innate immune responses by mRNAs may lead to side effects, including the production of unwanted proinflammatory cytokines that cause toxicity, as well as mRNA degradation and protein translation inhibition by antiviral molecules such as RNA-dependent protein kinase (PKR) and RNase [209, 247, 248]. Researchers have improved purification strategies and explored nucleoside modifications to mitigate undesired immune activation and enhance translation efficiency. Clinically approved mRNA vaccines employ purification techniques, such as high-performance liquid chromatography (HPLC), cellulose adsorption, and other protocols, to remove dsRNA contaminants. These vaccines also incorporate a Cap1 structure (m⁷GpppNm) at the 5’ end of the mRNA, which reduces RIG-I recognition. Additionally, mRNAs containing pseudouridine exhibit enhanced translational efficiency and reduced immunogenicity, as evidenced by lower serum levels of IFN-α in murine models [207–209]. Importantly, N1-methylpseudouridine (m1ψ) modification has been successfully incorporated into two approved COVID-19 mRNA vaccines, namely, mRNA-1273 (Moderna) and BNT162b2 (Pfizer-BioNTech).

Base-modified mRNAs with reduced immunogenicity have been highly successful in prophylactic antiviral vaccination, which requires high protein production and robust antibody responses. However, owing to immune-suppressive mechanisms and suboptimal T-cell priming in cancer settings, cancer vaccination requires more robust Th1-prone inflammatory responses to induce potent CTL responses. In this context, nonimmunogenic base-modified mRNAs may not be ideal for stimulating antitumor T-cell responses. Some studies have suggested that unmodified mRNA or the deliberate addition of dsRNA mimics can induce comparable or superior antitumor responses in murine tumor models [49, 249]. Thus, there is no consensus on which type of mRNA offers better antitumor efficacy, and both base-modified and unmodified mRNAs are being tested in clinical trials for cancer vaccination.

In addition to the immunogenic properties of mRNA itself, the nanocarriers used for delivery can also act as adjuvants, contributing synergistically to innate immune activation. Therefore, mRNA-based vaccine platforms must carefully balance the combined immunostimulatory effects of mRNAs and nanocarriers to elicit the desired Th1-skewed immune response while minimizing excessive innate activation, which could result in systemic toxicity. In the following sections, we will examine the adjuvant effects of several mRNA cancer vaccine platforms currently under clinical evaluation, with a focus on the synergistic roles of mRNA molecules and their delivery vehicles in shaping innate immune responses.

#### Adjuvant effect of RNA-LNPs

Empty LNPs possess self-adjuvant activity. For example, when empty LNPs are mixed with antigen proteins, robust antigen-specific T follicular helper (Tfh) cell and germinal center (GC) B-cell responses are elicited following a single intramuscular (i.m.) immunization [250]. In this study, the adjuvant activity of empty LNPs relied on the induction of interleukin-6 (IL-6) and the use of the ionizable lipid DLinDMA. The adjuvant effect of empty LNPs did not rely on the MyD88 or MAVS signaling pathways, as Tfh and GC B-cell responses remained intact in *Myd88–/–* or *Mavs–/–* mice [250]. In another study, LNPs containing heterocyclic ionizable lipid head groups activated the STING signaling pathway, enhancing the immunostimulatory effects on APCs in LNs and improving antitumor efficacy upon subcutaneous (s.c.) administration [251]. Moreover, C1 lipid-like LNPs act as self-adjuvants by triggering TLR4 signaling, promoting DC activation, and inducing the expression of proinflammatory cytokines such as IL-6, IL-12, and IL-1β [252]. These findings highlight the dual functionality of LNPs as both delivery vehicles and self-adjuvants, wherein ionizable lipids play a pivotal role in activating distinct pattern recognition receptor pathways. However, repeated LNP administration can induce anti-PEG antibody production, leading to high systemic reactogenicity [253].

The innate immune responses triggered by mRNA vaccines are crucial for eliciting robust antigen-specific adaptive immune responses. The mechanisms underlying the innate and adaptive immunity of the COVID-19 vaccine BNT162b2, which consists of m1ψ nucleoside-modified mRNAs encapsulated in LNPs, are well characterized [254]. BNT162B2-induced antibody and T-cell responses do not depend on multiple canonical innate immune pathways, including TLR2/3/4/5/7 signaling, the cGAS-STING pathway, NLRP3-dependent inflammasome activation, nor on necroptosis and pyroptosis. Notably, MDA5-mediated type I IFN signaling plays a crucial role in activating innate immune cells and promoting the expansion and effector functions of antigen-specific CD8^ +^ T cells [254]. The activation of MDA5 may be due to the spontaneous formation of dsRNA structures in the RNA-LNP vaccine.

#### Adjuvant effects of RNA-LPX and RNA-LPA

Unmodified RNA-LPXs are widely used in cancer vaccines. LPX-encapsulated unmodified mRNA but not LPX alone promoted IFNα production and enhanced the maturation and activation of splenic DCs [242]. However, m1Ψ-modified RNA-LPX is noninflammatory and can induce immune tolerance by expanding antigen-specific CD4 regulatory T cells [255]. These findings suggest the need to adjust the adjuvant effect of mRNA on the basis of the delivery system. Unmodified RNA-LPX induced TLR7-mediated biphasic IFN-α production by plasmacytoid dendritic cells (pDCs) and macrophages as early as 3 hours after i.v. injection [242]. In this context, the IFN-α response is essential for antigen-specific CD8^+^ T cells to acquire effector functions. The incorporation of unmodified RNA into RNA-LPAs also increased the induction of cytokines and chemokines, including type I/II IFNs, IL-12, CCL2, CCL4, and CXCL9. However, the potent innate stimulatory activity and antitumor ability of RNA-LPA depend on RIG-I but not TLRs or MDA5 [244].

To ensure safety, mRNA vaccines must achieve a balance between immunogenicity and reactogenicity, moderately stimulating the innate immune system to avoid excessive inflammation [246]. The toxic effects associated with RNA-LPX arise from its ability to stimulate IL-1β production, which subsequently triggers the release of a broad spectrum of proinflammatory cytokines [256]. The production of IL-1β may be dependent on TLR7/8 recognizing unmodified mRNAs to synthesize pro-IL-1β, as well as liposomes that activate the NLRP3 inflammasome to cleave pro-IL-1β. Humans exhibited greater sensitivity to RNA-LPX than laboratory mice because mice can upregulate anti-inflammatory IL-1ra, which competes with IL-1β for binding to IL-1R1. However, m1ψ-modified RNA-LPX failed to generate IL-1β and related cytokines in human PBMCs in vitro. This study also demonstrated that the toxicity of modified RNA-LNPs is influenced by the ionizable lipids used in their formulation. For example, modified RNA formulated in SM-102 LNPs induced robust IL-1β production and cytokine release, whereas formulation in MC3 LNPs resulted in weaker immune stimulation. Despite the toxicity associated with IL-1β, its immunogenic properties contribute to the expansion of antigen-specific T cells, as demonstrated in *IL-1ra*-deficient mice [256].

In summary, the components of nanoparticles, base modifications, and secondary structures of mRNA molecules can influence innate immune responses via various signaling pathways. The adjuvant activity of mRNA vaccines in clinical settings depends on both the chemical modification of the mRNA, which modulates recognition by innate immune sensors, and the nature of the lipid carrier used for delivery. In LNP formulations, mRNA is encapsulated within the core and remains inaccessible to membrane-bound innate immune sensors until it is released into the cytosol, where it is recognized primarily by cytosolic receptors such as RIG-I and MDA5. In contrast, in formulations such as LPX, mRNA is complexed on the surface of lipid particles, making it readily accessible to endosomal TLRs. However, how to modify the adjuvant effects of both RNA and nanoparticles to synergistically enhance DC activation and facilitate T-cell priming while minimizing side effects still needs to be further explored. This phenomenon is further complicated by the fact that several innate immune signaling pathways may lead to mRNA degradation and reduced antigen production.

### Toward next-generation mRNA vaccines

Several synergistic approaches have been explored to improve next-generation mRNA vaccines: optimizing LNP formulations to increase delivery efficiency and immunomodulatory functions, engineering mRNA structural elements to maximize protein expression, and incorporating mRNAs encoding immune-stimulatory adjuvants to amplify adaptive immunity.

#### Novel LNP formulation

Novel LNP formulations have been designed for targeted mRNA delivery, which reduce nonspecific toxicity effects and improve mRNA expression. Conventional LNPs exhibit significant liver accumulation, which may lead to undesired antigen expression in the liver. Adjusting the LNP composition enables selective delivery to target tissues. By screening LNP components, an endogenous lymph node-targeting LNP named 113-O12B, which can transfect DCs and macrophages and elicit better CD8^+^ T-cell responses following subcutaneous injection, was explored [257]. Another study achieved lung-, spleen-, and liver-targeted delivery by adding different selective organ-targeting molecules (SORTs) to conventional LNPs [258]. In addition to nonspecific organ accumulation, current LNP formulations exhibit low endosomal escape efficiency and induce multiple undesired inflammatory responses [259–261]. Individual LNP components—including ionizable lipids, helper lipids, sterols, and PEG lipids—can be substituted with novel molecules to improve mRNA expression or modulate innate inflammatory pathways [262]. For example, poly(carboxybetaine) (PCB) lipids have been used in place of PEG lipids and result in increased transfection and mRNA expression due to enhanced membrane fusion and endosomal escape, resulting in a better safety profile with lower inflammatory cytokine induction and limited antipolymer antibody responses [263]. Ionizable lipids are the major component affecting endosomal escape efficacy and are largely responsible for LNP-associated toxicity. Researchers have reported that ionizable lipids, which promote increased mRNA expression and endosomal escape, typically cause more severe inflammatory responses by generating large, irreparable endosomal holes recognized by cytosolic galectins. These galectins bind inner-membrane sugars and trigger downstream inflammation. Strikingly, rapidly biodegradable ionizable lipids preferentially create smaller endosomal holes that are reparable by the ESCRT pathway, thereby reducing inflammatory responses while preserving high mRNA expression [264]. Another study screened an ionizable lipid library and identified a lipid with an unsaturated tail, a dihydroimidazole linker, and cyclic amine head groups that activate the STING pathway when formulated in LNPs, resulting in increased antitumor efficacy in the context of mRNA vaccination [251]. Other components, such as cholesterol and helper lipids, can also be replaced with analogs to increase mRNA expression [265, 266]. However, significant challenges remain in systematically evaluating the safety profiles of these novel LNP components in humans, and clinical standards for assessing their safety have yet to be established.

#### Designing novel mRNA molecules

Extensive research has focused on enhancing the translational efficacy of mRNA vaccines. Key strategies include nucleotide modification, machine learning-guided mRNA design, and the exploration of noncanonical structures, such as circular and self-amplifying mRNAs [49, 217, 227]. Novel chemical synthesis methodologies are also being leveraged to design next-generation mRNA vaccines. Chen et al. developed a chemically synthesized branched poly(A) tail. The branch termini were modified with phosphorothioate (PS) and 2’-methoxyethyl (2MOE) groups to confer resistance to exonuclease degradation. This modified mRNA exhibited enhanced binding to cytoplasmic poly(A)-binding proteins (PABPCs), significantly prolonging protein expression duration and increasing expression levels [267]. The same group also engineered chemical modifications within the 5ʹUTR and cap structure. They introduced locked nucleic acid (LNA) and 2ʹ-O-methylation (2ʹOMe) modifications and created a branched 5’ structure by chemically linking two LNAm⁷G caps. These modifications collectively confer resistance to the decapping enzyme hDcp2 and increase the valency of eIF4E binding, resulting in improved protein translation [268]. These chemical innovations, which target both the 5ʹ and 3ʹ mRNA regions, illuminate pathways for designing mRNAs with increased antigen production in vivo. Furthermore, basic research into the intracellular fate of exogenously delivered mRNAs offers crucial insights for future vaccine design. A recent study revealed that Moderna’s mRNA-1273 features a 100-nt poly(A) tail terminating in a mΨCΨAG sequence. This structure enables readenylation by the TENT5A poly(A) polymerase in macrophages, prolonging protein expression. This mechanism was absent in Pfizer-BioNTech’s BNT162b2, which possesses a composite poly(A) tail (30A-10 N-70A) [269]. These findings reveal a novel mechanism for mRNA persistence in vivo and inform the design of future mRNA constructs.

#### Addition of adjuvant mRNA molecules

Beyond structural modifications to increase protein expression, incorporating adjuvant mRNAs encoding immune modulatory molecules (e.g., cytokines and innate immune activators) is an emerging strategy to increase vaccination efficacy. Peng et al. screened and identified IL-12 as a potent T-cell-stimulating cytokine that synergizes with an mRNA cancer vaccine. To mitigate the peripheral toxicity of IL-12, they tethered IL-12 to the surface of APCs by linking to a transmembrane domain. This design reduced systemic toxicity while preserving antitumor efficacy. Specifically, they reported that a preeffector T-cell population was induced by membrane-bound IL-12 and differentiated into highly potent effector T cells for enhanced tumor control [270]. Similarly, another study corroborated that IL-12 enhances mRNA vaccine-induced T-cell responses by driving effector T-cell differentiation [271]. IL-27, which acts downstream of mRNA vaccine-induced type-I interferon signaling, was found to directly support CD8⁺ T-cell expansion [272]. IL-27 mRNA-LNPs enhanced the proliferation of antigen-specific CTLs without altering their differentiation state, highlighting their potential as direct adjuvants to increase cytolytic CD8⁺ T-cell responses. *Zhivaki* et al. reported an mRNA encoding a self-DNA-reactive cGAS variant (cGASAN) that produces cGAMP upon binding intramitochondrial DNA. When delivered to DCs, this adjuvant mRNA upregulated lymph node-homing receptors, enhanced antigen presentation and costimulatory signaling, and promoted proinflammatory cytokine secretion, collectively contributing to stronger T-cell responses [273]. These studies highlight the potential of incorporating mRNA vaccines with adjuvant mRNA sequences encoding immune-stimulating molecules.

#### Balancing the adjuvant effect and antigen translation

One of the major challenges in developing mRNA vaccines is to moderate the activation of the innate immune system, which can exert both beneficial and detrimental effects [274]. mRNA vaccination leads to the early production of type I IFNs, which can increase the maturation and activation of APCs to promote T-cell priming and activation [275] or directly modulate T-cell responses [276]. However, type I IFNs simultaneously upregulate RNA-dependent protein kinase (PKR) and oligoadenylate synthetase (OAS), which inhibit mRNA translation and mediate mRNA degradation. Studies have shown that type I IFN signaling impairs the ability of mRNA-LPX vaccines to elicit CTL responses following subcutaneous, intradermal, and intranodal administration [277]. Similarly, in self-amplifying RNA (saRNA) LNP vaccines, type I IFNs reduce antigen expression and dampen CTL responses [278]. However, other studies have demonstrated that although type I IFNs reduce antigen expression, they can support T-cell responses when mRNA-LNPs are administered intramuscularly [254, 279] or when negatively charged mRNA-LPXs are delivered intravenously [242, 280]. In these cases, knockout or blockade of the type I IFN receptor (IFNAR) results in decreased quantity and cytotoxic ability of antigen-specific T cells. One key factor contributing to the divergent effects of type I IFNs is the route of administration [276]. Following subcutaneous injection of mRNA-LPX, type I IFN signaling impairs the induction of antigen-specific T cells, whereas it enhances T-cell responses following intravenouse administration. This discrepant role of type I IFN in T-cell activation may be attributed to the relative timing of the activation of IFNAR and TCR signaling in T cells. Additionally, the type of mRNA may also determine the effect of type I IFNs. SaRNAs are engineered for self-replication, enabling prolonged antigen expression at low microgram doses. However, this also increases its susceptibility to degradation and sensitivity to type I IFNs [281]. Furthermore, self-replication generates dsRNA intermediates, which are potent inducers of type I IFNs. To counteract this, encoding virus-derived innate inhibitory proteins—such as E3L from vaccinia virus, NS1 from influenza A virus, or the leader protein from cardioviruses—into saRNA vaccines has been proposed to suppress interferon signaling and enhance mRNA translation [281–285]. Notably, most studies assessing the role of type I IFNs in mRNA vaccine efficacy have employed high-affinity model antigens, such as ovalbumin (OVA), which may be less dependent on antigen expression levels and cannot simulate clinical scenarios. Future research should investigate how innate immune responses affect the efficacy of cancer mRNA vaccines across various nanoparticle formulations, routes of administration, and RNA platforms, particularly in the context of low-affinity tumor antigens.

Owing to immune-suppressive mechanisms and suboptimal T-cell priming in cancer, effective vaccination requires robust innate immune activation to generate potent CTL responses. As previously noted, potent adjuvants such as STING agonists are commonly used in conjunction with mRNA vaccines. However, careful balancing of agonist dosage and the mRNA payload is essential to prevent excessive type I IFN responses, which can complicate clinical evaluation [286, 287]. Therefore, adjuvant strategies incorporating mRNA-encoded specific cytokines or costimulatory molecules, which enhance T-cell responses without suppressing antigen translation, represent a preferable approach. Further research should explore novel mRNA vaccines that evade innate immune-driven side effects and elucidate the mechanisms underlying mRNA vaccine-induced T-cell activation. This knowledge will facilitate the rational design of vaccines capable of optimally orchestrating innate and adaptive immunity for cancer treatment.

### Clinical trials

In recent years, the development of cancer mRNA vaccines has been encouraging, with some clinical results advancing to phase III (Table 1) [288, 289]. In this section, we discuss the reasons for the failure of early clinical trials, highlight promising ongoing studies, and discuss the challenges faced by mRNA cancer vaccines.

#### Early clinical trials

One of the earliest clinical trials involved the direct injection of autologous amplified total tumor-derived naked mRNA combined with GM-CSF into stage III/IV metastatic melanoma patients [290, 291]. However, this phase I/II study did not demonstrate clinical efficacy, despite the observation of antitumor humoral immune responses in some patients. The lack of clinical effectiveness may be attributed to the heterogeneous amplification of total tumor mRNA in vitro, leading to the loss of lower-abundance mRNAs that encode tumor antigens [292]. Additionally, the insufficient expression and presentation of tumor antigens, likely due to competition with nonimmunogenic RNA-coding proteins, may have contributed to the failure. Therefore, subsequent clinical trials focused on selected TAAs or TSAs. Several early clinical trials utilizing unmodified naked tumor-antigen mRNA vaccines demonstrated their feasibility and safety, inducing antigen-specific CD4^+^ and CD8^ +^ T-cell responses [293, 294]. However, naked mRNA vaccines are unstable, which encourages the development of formulated vectors. Early trials focused primarily on protamine–mRNA complexes. Four protamine-mRNA vaccines—CV9103 [291], CV9104 [295], CV9201 [296], and CV9202 [297]—were designed to encode several TAAs for the treatment of advanced castration-resistant prostate cancer or non-small cell lung cancer (NSCLC), with CV9202 being combined with local radiation therapy. The overall survival (OS) benefits of these protamine-mRNA vaccines have been limited. The lack of clinical responses may be attributed to several factors, including suboptimal mRNA modification and delivery, central immune tolerance to the selected TAAs, the highly immunosuppressive microenvironment in advanced-stage tumors, and insufficient induction of long-term memory responses required for durable clinical benefit. These challenges underscore the need for optimized tumor antigen selection, more efficient delivery systems, and potential combination therapies.

#### TAAs cancer mRNA vaccines

Nonformulated mRNAs are easily degraded by extracellular RNases. Consequently, formulated LPX and LNP-encapsulated mRNA vaccines are frequently utilized in ongoing clinical trials. FixVac (BNT111) is an intravenously administered RNA-LPX vaccine encoding four full-length melanoma TAAs—NY-ESO-1, MAGE-A3, TPTE, and tyrosinase (NCT02410733, NCT04526899) [37]. The administration of FixVac alone or in combination with anti-PD1 therapy induced durable objective responses in patients with stage IIIB-C and IV melanoma, with mild to moderate adverse events. In a phase I study, circulating antigen-specific T cells were expanded by several orders of magnitude following a repeat boost strategy, and these cells were fully functional, suggesting that selective TAAs may disrupt immune tolerance. More than 75% of the analyzed patients developed immune responses against at least one TAA, particularly NY-ESO-1 and MAGE-A3. Additionally, vaccine-induced T cells in some patients were sustained for over one year, which may contribute to long-term clinical benefits. FixVac combined with PD1 blockade was more effective than monotherapy (partial response rate: 12% vs. 35%). These findings support the potential of combining mRNA vaccines with ICB therapy for patients with low mutational burdens. A phase II trial of FixVac combined with cemiplimab is ongoing. RNA-LNP vaccines are also designed to target and eliminate immunosuppressive regulatory cells as well as cancer cells, thereby mitigating the immunosuppressive TME and enhancing T-cell effector functions. For example, mRNA-4359, which encodes both PD-L1 and indoleamine 2,3-dioxygenase 1 (IDO1) antigens, is being evaluated in patients with advanced solid tumors [298].

#### TSAs as cancer mRNA vaccines

It is very convenient to include multiple minigenes encoding mutated antigens in mRNA vaccines. BNT122 is a personalized RNA-LPX vaccine containing up to 20 neoepitopes presented on MHC class I and MHC class II molecules [299]. In a phase I study (NCT04161755), patients with surgically resected pancreatic ductal adenocarcinoma (PDAC) were sequentially treated with anti-PD-L1, BNT122, and a modified four-drug chemotherapy regimen (mFOLFIRINOX). The combination treatment is well tolerated and induces de novo, high-magnitude neoantigen-specific T-cell responses in 50% of patients (8/16). Half of these T-cell responses targeted more than one vaccine neoantigen. Moreover, over 80% of these specific T cells were detectable up to three years after administration in responders, with 75% of responders remaining disease free. These results suggest that neoantigen-based mRNA vaccines can generate potent T-cell responses and long-lived T cells for tumor control [300]. A phase II trial of BNT122 is ongoing (NCT04161755). mRNA-4157 (V940) is another individualized, nucleoside-modified RNA-LNP vaccine encoding up to 34 melanoma neoantigens that is administered via intramuscular injection [301]. In a phase 2 trial, mRNA-4157, with or without pembrolizumab (anti-PD-1), was used to treat patients with completely resected melanoma (stage IIIB-IV). The 2.5-year overall survival (OS) and recurrence-free survival (RFS) rates for the combination treatment were 96% and 74.8%, respectively, whereas they were 90.2% and 55.6%, respectively, for pembrolizumab alone. Ongoing phase III clinical trials are evaluating mRNA-4157 in combination with pembrolizumab as adjuvant therapy for patients with resected high-risk melanoma (NCT05933577) and non-small cell lung cancer (NSCLC) (NCT06077760), making mRNA-4157 the first cancer mRNA vaccine to reach phase III. However, despite the potential of neoantigens to generate tumor-reactive T cells with high affinity due to a lack of thymic negative selection, not all neoantigen-based mRNA vaccines tested in clinical trials have demonstrated clinical efficacy. For example, mRNA-4650, which encodes up to 20 neoantigens expressed by autologous tumors, failed to elicit objective clinical responses in patients with gastrointestinal cancer, potentially because of the lower responsiveness to vaccination caused by lymphodepletion during the TIL therapeutic regimen [302], which suggests the importance of appropriate schedules and combination therapies.

#### Self-amplifying mRNA cancer vaccines

In addition to nonreplicating mRNAs, self-amplifying mRNAs (saRNAs) have been utilized to increase protein expression levels. In a phase I study (NCT03639714), a heterologous prime/boost vaccination strategy was evaluated [303]. Specifically, heterologous chimpanzee adenovirus (ChAd68) for priming followed by neoantigen saRNA-LNP boosts, in combination with nivolumab and ipilimumab, were administered to patients with advanced metastatic solid tumors. This regimen was well tolerated and induced functional neoantigen-specific effector memory CD8^+^ T-cell responses in all patients. A phase II/III study has been initiated to further investigate this strategy in patients with metastatic colorectal cancer [304]. Moreover, an “off-the-shelf” vaccine utilizing this heterologous prime/boost regimen in combination with ICB therapy was assessed in a phase I trial (NCT03953235) [305]. This vaccine encodes 20 shared oncogenic mutations within a single vector, such as KRAS mutations (G12D, G12C, G12V, and G13D) and TP53 mutations, and is intended to circumvent the need for individualized neoantigen prediction. Despite the generation of antigen-specific T-cell responses in most patients, the overall response rate was 0%, with median recurrence-free survival (RFS) and overall survival (OS) rates of 1.9 and 7.9 months, respectively. Interestingly, T-cell responses were predominantly directed toward tumor-unrelated TP53 epitopes rather than the KRAS mutations present in the tumors, indicating the existence of a potential hierarchy of immunodominance in the processing and presentation of multiple epitopes within the same APCs. To address this issue, researchers have split mRNAs into separate vectors to ensure delivery to distinct APCs, and encoding repeated copies of each epitope could optimize KRAS presentation in mice [305]. An optimized shared vaccine encoding only four highly prevalent KRAS-derived mutations (each of which is repeated four times) is currently being evaluated in a phase II study (NCT03953235).

#### Combination therapy with CAR-T cells and RNA vaccine

Chimeric antigen receptor (CAR)-T cells have shown impressive efficacy in patients with B-cell malignancies, where CAR-T cells are easily expanded and reactivated by circulating B cells. However, transferred CAR-T cells targeting solid tumors show limited persistence and reactivation due to the lack of efficient in vivo stimulation. CAR-T-cell-enhancing mRNA vaccines have been designed to deliver CAR antigens for the expansion and reactivation of CAR-T cells in a proper environment inside lymphoid tissues. The CARVac is an RNA-LPX vaccine designed to deliver and induce the expression of CLDN6 on the surface of APCs within lymph tissues. The combination therapy of CLDN6-specific CAR-T cells with CARVac is currently undergoing phase I/II trials (NCT04503278) [306, 307]. CARVac significantly expanded the transferred CAR-T cells in secondary lymphoid tissues and had a superior antitumor effect on a mouse model. In a phase I study, the ORR was 38%, and the DCR was 69%, with CAR-T cells observed to persist beyond 90 days in some patients [308].

Although substantial progress has been made in the development of cancer mRNA vaccines, several challenges remain unresolved. First, balancing innate and adaptive immunity to achieve rapid and durable clinical effects remains a challenge. Unlike prophylactic vaccines, therapeutic cancer vaccines must induce a strong inflammatory environment to overcome peripheral and intratumoral immunosuppression for efficient antigen-specific T-cell priming. In this context, optimizing the adjuvant effect of cancer mRNA vaccines, which can induce a proinflammatory milieu without impairing mRNA translation, might be necessary. Compared with modified mRNAs, unmodified mRNAs may elicit equal or even more robust antitumor responses in some contexts [49, 309]. Systemic administration may elicit more rapid systemic innate immune responses; however, potential toxicity must be carefully considered. Second, most tumor antigens, particularly TAAs, are poorly immunogenic and often subject to immune tolerance [310]. Strategies to overcome immune tolerance and generate a large pool of antigen-specific effector T cells during the priming phase are essential. Third, while many clinical cancer mRNA vaccines encode both CD8^+^ and CD4^+^ T-cell epitopes, the observed immune responses are predominantly mediated by CD4^+^ T cells rather than CD8^+^ T cells [291, 294, 297, 302]. mRNA vaccines may preferentially elicit antigen-specific CD4^+^ T cells, potentially due to the broader clonal heterogeneity of CD4^+^ T cells. Questions remain regarding whether both CD8^+^ and CD4^+^ epitopes are necessary [220] and whether vaccine-induced CD4^+^ T-cell responses can promote CD8 ^+^ T-cell priming and differentiation for improved tumor control [311–313]. Both the adjuvanticity and the antigen hierarchy of mRNA vaccines require further investigation to optimize T-cell responses.

## Administration route

The route of administration significantly influences the innate and adaptive immune responses of different formulations of cancer vaccines. Therefore, selecting the appropriate administration route is critical and should be carefully evaluated to ensure successful vaccination outcomes.

### Peptide vaccines

The injection route can significantly influence both the immunogenicity and toxicity of peptide vaccines. Common injection routes include s.c., i.m., and i.v. injections. I.m. injections are commonly used because of their ability to administer larger vaccine volumes and their favorable safety profile. S.c. injections, while facilitating more efficient vaccine drainage into lymph nodes through a dense network of lymphatic vessels, are limited by the smaller volume that can be injected. I.v. injections allow for systemic exposure of vaccines to lymphoid organs but may also induce systemic side effects [314, 315].

Hussein et al. compared T-cell responses associated with different injection routes using TriVax (which contains a palmitoylated peptide, anti-CD40 monoclonal antibody, and poly-ICLC) and reported that i.v. injection generated substantially greater CTL responses than i.m. and s.c. injections did [315]. I.v. injections may promote systemic antigen uptake by a larger number of APCs, which allows for more efficient recruitment and activation of antigen-specific CTL precursors. However, systematic exposure to adjuvants may increase toxicity and limit clinical applicability. Another study assessed a self-assembling peptide conjugated to a TLR7/8 agonist (SNP-7/8a) and reported that the same vaccination dose generated fewer antigen-specific T cells via the i.v. route (SNP-IV) than via the s.c. route (SNP-SC). Interestingly, the antigen-specific T cells generated after SNP-IV vaccination retained more Tcf1^+^ stem-like cells. When the vaccination dose for SNP-IV was increased to induce a comparable number of T cells to SNP-SC, the SNP-IV-generated T cells exhibited enhanced synergy with checkpoint blockade therapy and resulted in better tumor control. This study revealed that the duration of antigen presentation by DCs was critical in shaping the quantity and quality of CD8^+^ T-cell responses, with SNP-IV leading to transient antigen presentation by DCs, fostering the generation of stem-like T cells [316].

Intranodal (i.n.) injection is less commonly employed because of the complexity of the injection process and volume limitations. Nonetheless, studies have demonstrated that, compared with traditional vaccination routes, a dose of 10^6^-fold reduction in antigen and 100-fold reduction in adjuvant achieves enhanced protection and fewer side effects in i.n. injections [317, 318]. Mechanistic studies on i.n. injection might inspire new designs of vaccines to mimic this microenvironment.

### mRNA vaccines

The route of administration significantly influences the organ distribution of nanoparticles, the types of immune cells that take up mRNA, the kinetics of antigen expression, and the resulting local or systemic immune responses, all of which influence the therapeutic efficacy of cancer mRNA vaccines. Common administration routes include i.m., intradermal (i.d.), s.c., and i.v. injections. The choice of the administration route is typically determined by the characteristics of the delivery nanoparticles and the desired immune response, whether localized or systemic.

#### Local administration

Skin and muscle tissues contain abundant resident APCs, and innate immune cells, which can take up RNA-LNPs locally and then migrate to dLNs after s.c. or i.m. vaccination. Furthermore, lymphatic vessels in these tissues facilitate the direct flow of RNA-LNPs into dLNs, where they transfect resident APCs [319, 320]. Intramuscular injection has been widely employed during the administration of COVID-19 mRNA vaccines. In murine and nonhuman primate (NHP) models, following i.m. injection, RNA-LNPs are highly taken up by stromal cells at the injection site, including endothelial cells, pericytes, and fibroblasts, as well as by myeloid cells such as macrophages, neutrophils, mast cells, and DCs [279, 319, 321]. Fibroblasts express IFNβ and chemokines in response to RNA-LNPs, which recruit inflammatory immune cells to the injection site [279]. The mRNA-encoded protein is expressed primarily in macrophages, fibroblasts, and adipocytes at the injection site, whereas it is highly expressed in macrophages, DCs, and monocytes in dLNs [254, 279, 319, 321]. Although RNA-LNPs are located primarily in the injection site and dLNs, mRNA concentrations have also been detected in the spleen, liver, and blood [319, 322]. Consistently, protein expression was detected as early as 4 hours postadministration at both the injection site and dLNs in nonhuman primates (NHPs). The skin harbors many epidermal Langerhans cells, dermal DCs, and macrophages, which suggests that i.d. delivery may elicit stronger immune responses and prolong protein expression [321]. However, most studies have shown comparable immune responses following i.d., i.m., and s.c. vaccinations [323]. Additionally, owing to technical difficulties and increased adverse effects, i.d. and s.c. injections are used less frequently than i.m. injections [324].

#### Systemic administration

Systemic injection of RNA vaccines allows circulation to the spleen, where abundant antigen-presenting cells, particularly cDC1s, are present. Compared with s.c. injection, intravenous injection of spleen-targeting RNA-LPX elicits a stronger immune response and induces a greater proportion of antigen-specific CD8^ +^ T cells [242]. In contrast, RNA-LNP-based cancer vaccines are less commonly administered via i.v. injection because of liver accumulation, which may promote antigen presentation in an immune-tolerant microenvironment [322, 325]. However, modified LNP formulations designed for spleen-targeted delivery of mRNAs, such as SORT LNPs [258] and antibody/peptide-modified LNPs [326, 327], have been developed, although their clinical exploration for cancer vaccines remains limited.

The kinetics of induced proinflammatory cytokine release could be influenced by the RNA vaccine injection route, which affects the induction of the antigen-specific T-cell response. Studies suggest that different administration routes may induce opposing effects of type-I IFN signaling on T-cell responses. The induction of type-I IFN promoted antigen-specific T-cell responses when mRNA-LPX was i.v. injected but suppressed T-cell responses when the vaccine was delivered s.c. or i.d [276, 277]. A potential explanation for these opposing effects is the relative timing of the activation of IFNAR and TCR signaling in T cells. When IFNAR signaling coincides with TCR stimulation, type I IFNs promote effector T-cell responses. In contrast, IFNAR signaling preceding TCR engagement may lead to the formation of different STAT complexes, contributing to an inhibitory effect [328, 329]. Thus, the route of administration likely influences the kinetics of T-cell exposure to type I IFN and TCR engagement, ultimately impacting vaccination outcomes. However, a comprehensive understanding of the immune responses elicited by different administration routes and a direct comparison of their antitumor effects remain insufficiently explored.

## Concluding and future perspectives

Early cancer vaccines against TAAs have shown limited therapeutic efficacy because of antigen immune tolerance and the lack of a potent adjuvant and delivery system. Recent therapeutic cancer vaccines have undergone a remarkable transformation, driven by advances in antigen identification, delivery platforms, and immune modulation strategies. Advancements in next-generation sequencing technology, computational capabilities, and predictive algorithms informed by the accumulation of wet-laboratory data have facilitated the identification of nontolerant neoantigen candidates for cancer vaccination. Furthermore, novel delivery platforms, including peptide-based vaccines and RNA vaccines, have demonstrated the ability to prime potent CD4^+^ and CD8 ^+^ T-cell responses, not only against neoantigens but also against TAAs. Despite these achievements, several challenges may impede the clinical efficacy of cancer vaccines.

### Identifying shared neoantigens

Current neoantigen-based cancer vaccines require highly personalized antigen identification and vaccine formulation, resulting in formidable medical costs that are inaccessible to many cancer patients. Although some clinical trials have demonstrated the efficacy of TAA-based vaccination with novel platforms, the risk of autoimmune diseases poses a significant barrier, particularly for cancer types in which autoimmunity could have fatal consequences. Identifying shared tumor-specific antigens may address these limitations. A substantial proportion of neoantigens have been found to originate from noncoding and “dark matter” regions of the genome, as well as from noncanonical translation processes, which need further exploration for shared epitopes. Further advancements in NGS technology, mass spectrometry-based MHC ligandome analysis, and novel computational algorithms could unveil those antigens that can truly activate patients’ T cells, enabling their evaluation in clinical studies.

### Opportunities in other vaccine platforms

In addition to peptide- and mRNA-based cancer vaccines, several other vaccine platforms have been developed for cancer immunotherapy, including DC vaccines, in situ vaccines, viral vector-based vaccines, and bacteria-based vaccines. DC vaccines have been extensively explored in the clinic for cancer vaccination and exhibit good tolerance and elicit antigen-specific T-cell responses. Several excellent reviews have provided valuable insight into this vaccination platform [330–333]. In situ vaccines aim to increase antigen release from tumor cells at the tumor site to elicit adaptive immune responses without the need for prior identification of tumor antigens [334]. Key determinants of in situ vaccine efficacy include the adequate release of tumor antigens from cancer cells and the induction of robust antigen processing by APCs through the activation of immunogenic cell death (ICD) and innate immune responses [335]. Thus, various strategies have been employed to enhance the ICD and antigen processing. These include intratumoral injection of chemotherapeutic hydrogels for local drug retention and sustained release [336, 337]; the design of nanosensitizers for radiotherapy, photodynamic therapy, or sonodynamic therapy [338–343]; and the codelivery of pattern recognition receptor (PRR) agonists [338, 344] or ICD inducers [345]. Additionally, the intratumoral administration of oncolytic viruses has emerged as a promising in situ vaccination approach, offering advantages such as selective replication within tumor cells, direct oncolysis, and the activation of innate immune-sensing pathways [346, 347]. Current research is focused on genetically engineering oncolytic viruses to express tumor-irrelevant bystander epitopes [348], tumor-associated antigens, cytokines, or immune checkpoint inhibitors, thereby increasing their antitumor efficacy [349]. Viral vector-based vaccines are typically engineered by deleting pathogenic genes and incorporating genes encoding tumor antigens [350]. Through genetic modification, viral vectors can target APCs with high transduction efficiency while minimizing pathogenicity. Commonly used viral vectors in clinical studies include adenovirus (Ad), adeno-associated virus (AAV), herpes simplex virus (HSV), and retrovirus (RV) [351, 352]. Bacteria-based vaccines exploit the unique properties of bacteria, such as their intrinsic tumor-targeting capabilities, preferential proliferation in hypoxic and immunosuppressive TMEs, and strong stimulation of the innate immune system, which contributes to TME remodeling [353–355]. Engineered bacterial strains, such as *Listeria monocytogenes* [356, 357] and the probiotic *Escherichia coli* [358], have been developed to express and release high levels of tumor antigens following systemic administration. The integration of synthetic biology and other immunotherapeutic strategies with these vaccine platforms holds promise for broader clinical application.

### Combination therapy for optimized tumor control

The immunosuppressive TME poses a significant barrier to the infiltration, activation, proliferation, and effector functions of vaccine-induced T cells in tumors [359, 360]. Insufficient T-cell infiltration, driven by the lack of attractant chemokines and abnormal tumor vasculature, significantly limits the effectiveness of vaccine-induced T-cell trafficking into the TME. Moreover, infiltrated T cells often encounter immunosuppressive signals from myeloid cells, such as tumor-associated macrophages, which provide suboptimal TCR and costimulatory signals while expressing inhibitory surface markers and soluble factors that drive T-cell exhaustion [361, 362]. The scarcity of professional APCs within the TME and the lack of T-cell-supporting cytokines exacerbate these challenges, leading to limited T-cell proliferation, survival, and effector function. Combining cancer vaccines with other immunomodulatory therapies may help overcome these barriers and achieve optimal tumor control. Angiogenesis inhibitors can normalize the tumor vasculature, enhancing T-cell infiltration. Radiotherapy and in situ vaccination agents, such as STING agonists, TLR agonists, and oncolytic viruses, can create an inflammatory TME that supports T-cell infiltration while providing additional tumor antigens to enhance T-cell priming in a feed-forward loop [6]. Immune checkpoint inhibitors and tumor-targeting cytokines can further enhance T-cell survival and effector differentiation, as demonstrated in early-phase clinical trials. Owing to the heterogeneity of cancer, antigen-negative cell clones may be selected after vaccination, leading to tumor relapse. Combination therapies may also induce epitope spreading in T-cell responses, which broadens the T-cell repertoire and triggers additional tumor antigen-directed immune responses [149, 150]. Further investigation of the mechanisms of epitope spreading will help identify a more effective combination therapy strategy to completely control tumors.

### Identifying predictive biomarkers to predict patients’ response to vaccines

Identifying predictive biomarkers for selecting patients who will respond to cancer vaccine therapy is also essential. Early-phase clinical trials have shown that a subset of patients who receive neoantigen-based vaccination exhibit a response characterized by robust T-cell activation and prolonged survival [299]. However, it remains uncertain whether robust T-cell activation represents a direct survival benefit conferred by vaccination or is a biomarker reflecting preexisting antitumor immunity and patients’ responsiveness to vaccination. Clinical evidence suggests that while cancer vaccines targeting unselected patient populations may fail to produce statistically significant survival benefits, they may offer substantial advantages for subsets of patients with specific biomarkers or favorable physiological conditions [363].

In conclusion, while therapeutic cancer vaccines have yet to achieve their full clinical potential, the integration of novel antigen discovery pipelines, cutting-edge delivery technologies, and rational combinatory strategies could transform them into a cornerstone of cancer immunotherapy. The accumulated knowledge from preclinical and clinical research will help better understand the immune mechanism of cancer vaccines and design novel vaccine formulations for durable and effective tumor control.
